# Synthesis of spirocyclic scaffolds using hypervalent iodine reagents

**DOI:** 10.3762/bjoc.14.152

**Published:** 2018-07-17

**Authors:** Fateh V Singh, Priyanka B Kole, Saeesh R Mangaonkar, Samata E Shetgaonkar

**Affiliations:** 1 Chemistry Division, School of Advanced Sciences (SAS), VIT University, Chennai Campus, Chennai-600 127, Tamil Nadu, India

**Keywords:** hypervalent iodine reagents, iodoarenes, natural products, oxidative cyclization, spirocyclic compounds

## Abstract

Hypervalent iodine reagents have been developed as highly valuable reagents in synthetic organic chemistry during the past few decades. These reagents have been identified as key replacements of various toxic heavy metals in organic synthesis. Various synthetically and biologically important scaffolds have been developed using hypervalent iodine reagents either in stoichiometric or catalytic amounts. In addition, hypervalent iodine reagents have been employed for the synthesis of spirocyclic scaffolds via dearomatization processes. In this review, various approaches for the synthesis of spirocyclic scaffolds using hypervalent iodine reagents are covered including their stereoselective synthesis. Additionally, the applications of these reagents in natural product synthesis are also covered.

## Review

### 1. Introduction

**Figure 1:**
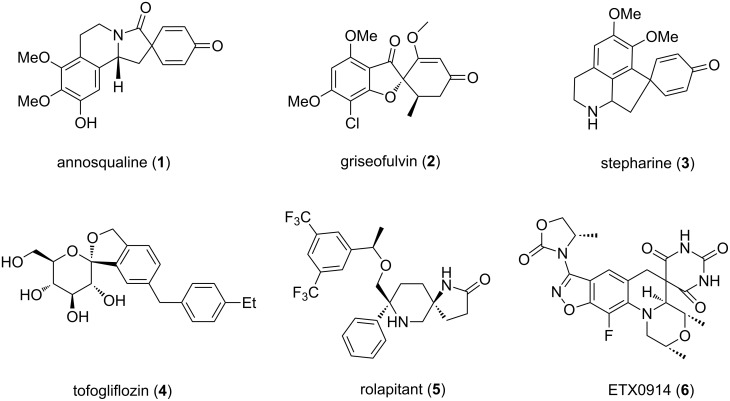
The structures of biologically active natural and synthetic products having spirocyclic moiety.

The chemistry of spirocyclic compounds is a well established research area of organic and medicinal chemistry [1–5]. These scaffolds are common structural motifs found in various classes of naturally occurring systems [6–8]. More importantly, various natural and synthetic products containing a spirocyclic ring are currently used as commercial drugs for the treatment of several health problems [9–10]. Annosqualine (**1**) is an isoquinoline-cored alkaloid and it was isolated in 2004 from the stem of *Annona squamosa* [11] (Figure 1).

Griseofulvin (**2**) is a spirobenzofuranone-based naturally occurring compound which was isolated from *Penicillium griseofulvum* in 1939 [12]. In 1959, it was launched in the market as antifungal agent for the treatment of ringworm in human beings and animals [4,13]. Stepharine (**3**) is a member of the proaporphine alkaloid family and isolated from an angiosperm *Stephania glabra* [14]. Tofogliflozin (**4**) is a synthetic spirocyclic glycoside that was launched as antidiabetic agent in 2012 in Japan [15]. Rolapitant (**5**) is a marketed drug that was approved in 2015 for the treatment of nausea and vomiting [16]. Compound **6** is a spiropyrimidinetrione analogue which is currently in clinical trials for the treatment of gonorrhea [17]. There are several ways available in literature for the synthesis of spirocyclic compounds but most of them are associated either with transition metals or hypervalent iodine reagents [1–3].

Hypervalent iodine reagents provide various functional group transformation opportunities in organic chemistry. Their environment-friendly nature and mild reaction conditions makes them more attractive candidates for the replacements of various toxic metals in organic synthesis [18–31]. These reagents are more popular for their oxidizing properties [32–38] and electrophilic nature of different iodine(III) reagents has been explored to developed various synthetic transformation including rearrangements [39–62]. Hypervalent iodine chemistry has now become a well-established research area and various book chapters [19–20,27] and review articles [21–24,31–35,60,63–64] appeared to explain the chemistry of these reagents. In the past two decades, a number of organic chemists used these reagents for the construction of a variety of spirocyclic scaffolds. In 2008, Quideau and co-workers published a nice review article where they have described various spirocyclization reactions using hypervalent iodine reagents via dearomatizations of aromatic phenolic species [32]. This review article is quite useful for readers who want to know the chemistry involved during the dearomatization of phenols and to find the relevant literature available until 2008. In this review article, various approaches for the synthesis of spirocyclic scaffolds using hypervalent iodine reagents are covered including stereoselective reactions.

**Scheme 1:**
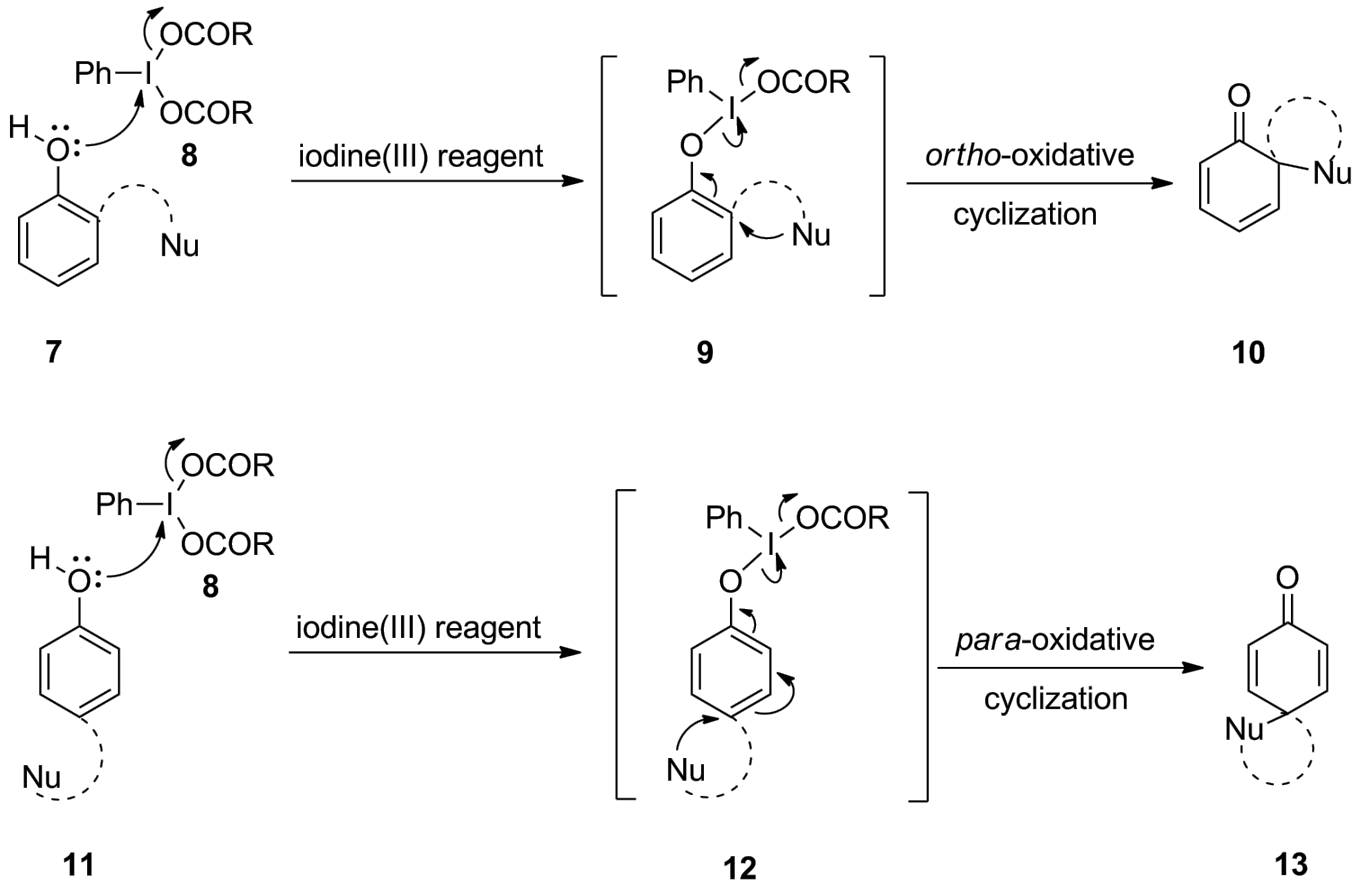
Iodine(III)-mediated spirocyclization of substituted phenols **7** and **11** to **10** and **13**, respectively.

Hypervalent iodine reagents are mainly popular for their oxidative properties but various iodine(III) reagents have been used as electrophiles. Numerous iodine(III) reagents have been successfully used to achieve diverse spirocyclic scaffolds. Phenols **7** or **11** having an internal nucleophile at *ortho-* or *para-*position can be used as starting material for the synthesis of *ortho*- and *para*-spirocyclic compounds in the presence of iodine(III)-based electrophiles (Scheme 1). Phenolic oxygen of compound **7** attacks to the iodine of **8** to form intermediate **9**. Furthermore, on nucleophilic attack of the internal nucleophile to the *ortho*-position intermediate **9** converts to *ortho*-spirocyclic compound **10** with the elimination of the hypervalent iodine moiety. Similarly, *para*-spirocyclic compounds **13** can be achieved starting from compounds **11** and iodine(III) reagent **8** (Scheme 1). The synthesis of spirocyclic compounds can be achieved using stoichiometric or catalytic amounts of iodine(III) reagents. According to literature reports, both heterocyclic and carbocyclic spirocyclic compounds can be achieved using these reagents [27,32].

### 2. Synthesis of spirolactones

#### 2.1. Using stoichiometric amounts of iodine(III) reagents

The history of the utility of hypervalent iodine reagents in the synthesis of spirocyclic compounds is going to become quite old now. Initially, iodine(III) reagents were applied for synthesis of spirocyclic in 1990s [65–66]. In 1991, Kita and co-workers [67] established the synthesis of spirohexadiones from *N*-acyltyramines using iodine(III) reagent. After these reports, numerous hypervalent iodine-mediated spirocyclizations were investigated and phenolic oxidations of substrates have been explored for the construction of spirodienone motifs [21,64].

**Scheme 2:**
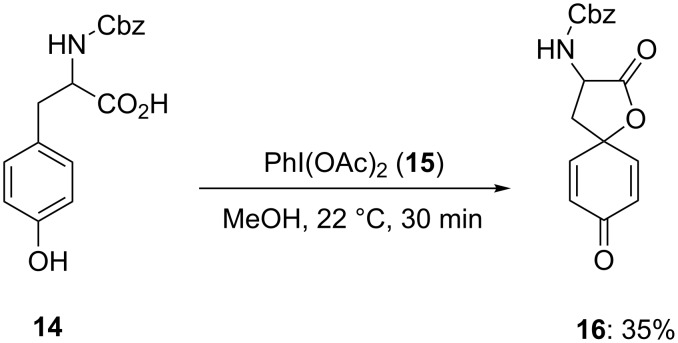
PIDA-mediated spirolactonization of N*-*protected tyrosine **14** to spirolactone **16**.

In 1993, Wipf and Kim [68] employed PIDA **(15**) for spirocyclization of N*-*protected tyrosine **14** to spirolactone **16**. The spirocyclization reaction was carried out in methanol using stoichiometric amounts of PIDA (**15**) and spirolactone **16** was isolated in 35% yield (Scheme 2). Probably, the cyclization reaction proceeded via dearomatizaion of phenolic substrate **14** followed by nucleophilic attack of the carbonyl moiety of carboxylic group.

**Figure 2:**
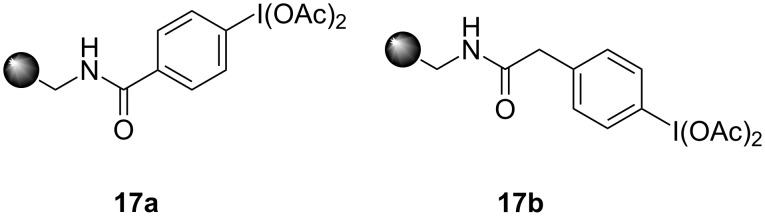
The structures of polymer-supported iodine(III) reagents **17a** and **17b**.

Furthermore, Giannis and co-workers [69] reported the synthesis of novel aminomethylpolystyrene-supported (diacetoxyiodo)benzene (PSDIB) reagents **17a** and **17b** starting from aminomethylated polystyrene with 4-iodobenzoic acid and 4-iodophenylacetic acid in two steps (Figure 2).

**Scheme 3:**
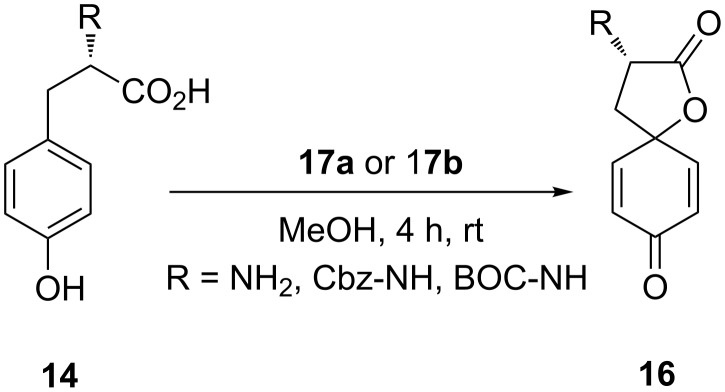
Spirolactonization of substrates **14** to spirolactones **16** using polymer-supported reagents **17a** and **17b**.

Both polymer-supported reagents **17a** and **17b** were used in similar spirocyclizations of tyrosine **14**. Both tyrosine **14a** and N-protected tyrosine derivatives **14b**,**c** were used as starting material and results of their spirolactonization are summarized in Table 1 (Scheme 3).

The spirolactonization products **16** were isolated in excellent yields when reactions were performed with substrates **14** (R = NH_2_) having free amino group (Table 1, entries 1 and 2). Notably, the poor yields were observed during the spirolactonization of N-protected tyrosine derivatives **14b** and **14c** (Table 1, entries 3–6). The advantage of this reaction is that the polymer-supported reagent can be regenerated and reused without loss of any significant activity [69].

**Table 1:** Spirolactonization of substrates **14** to spirolactones **16** using polymer-supported reagents **17a** and **17b**.

entry	substrate **14**	PS-iodine(III) reagent	**16** yields (%)

1	**14a**: R = NH_2_	**17a**	82
2	**14a**: R = NH_2_	**17b**	80
3	**14b**: R = Cbz-NH	**17a**	25
4	**14b**: R = Cbz-NH	**17b**	26
5	**14c**: R = Boc-NH	**17a**	24
6	**14c**: R = Boc-NH	**17b**	25

**Scheme 4:**
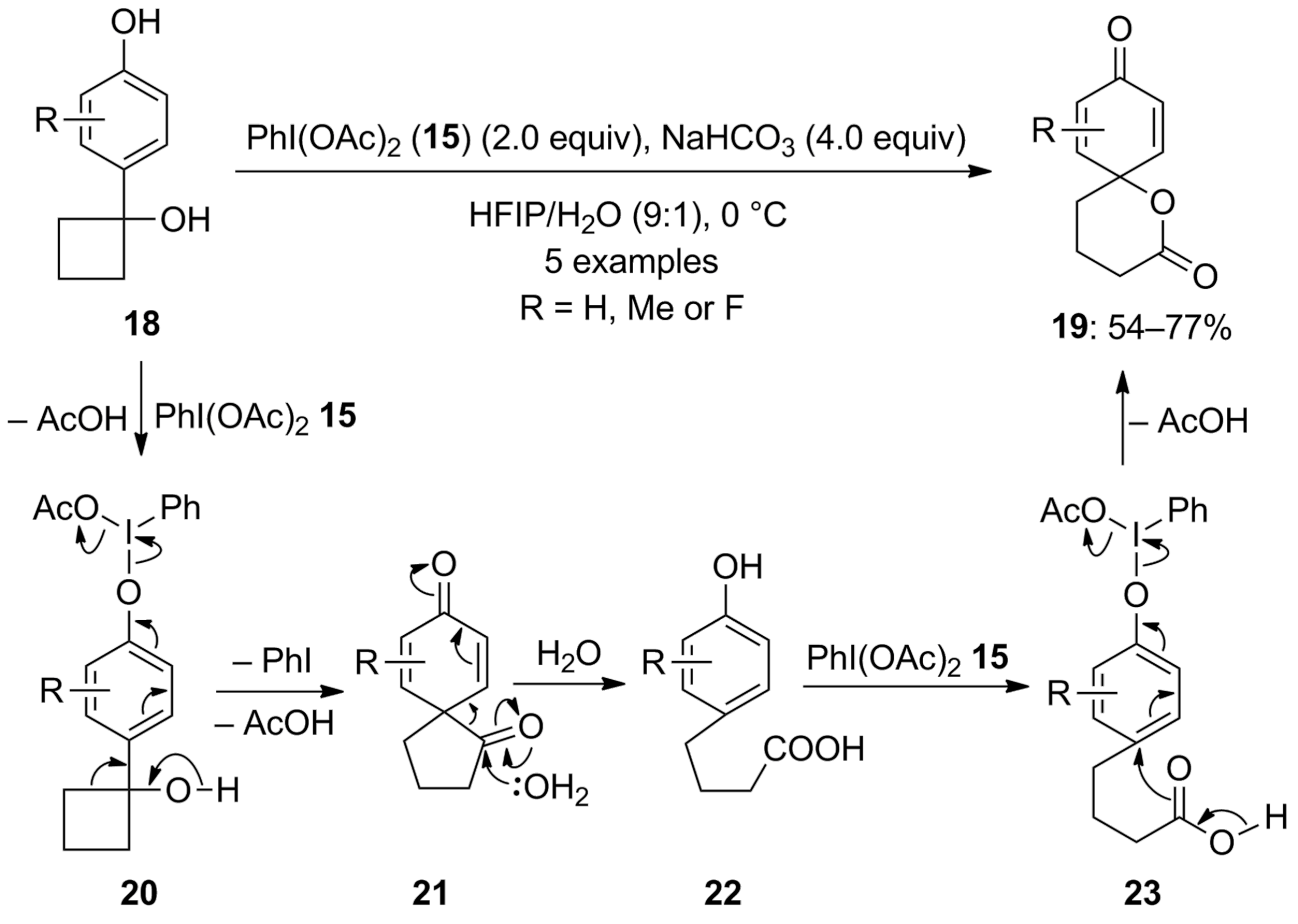
PIDA-mediated spirolactonization of 1-(*p*-hydroxyaryl)cyclobutanols **18** to spirolactones **19**.

In 2010, Kita and co-workers [70] developed another approach for PIDA-mediated spirolactonization of 1-(*p*-hydroxyaryl)cyclobutanols **18** to spirolactones **19** in good yields (Scheme 4). The reaction was initiated with formation of an intermediate **20** by the oxidation of the phenolic hydroxy group of **18**, which rearranged to compound **21**. Furthermore, water attacks the ketone moiety of **21** to form *para*-substituted phenol **22**. The phenolic intermediate **22** is further oxidized with another molecule of PIDA (**15**) to form intermediate **23**, which yielded the final product **19** on intramolecular cyclization [70].

**Scheme 5:**
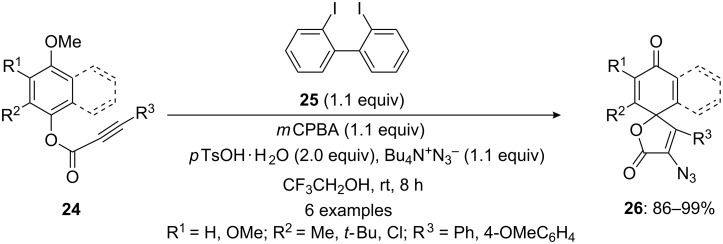
Iodine(III)-mediated spirocyclization of aryl alkynes **24** to spirolactones **26** by the reaction with bis(iodoarene) **25** in the presence of *m*CPBA.

Furthermore, Kita and his research group [71] reported an iodine(III)-mediated cyclization of arylalkynes **24** to spirocyclic products **26** by in situ-generated active hypervalent iodine species. In this report, *para*-substituted esters **24** were cyclized to corresponding spirolactones **26** using stoichiometric amount of bis(iodoarene) **25** with terminal oxidant *m*CPBA in the presence of TsOH·H_2_O in TFE (Scheme 5). In this reaction, active hypervalent iodine species was generated in situ by the oxidation of bis(iodoarene) **25** using *m*CPBA as terminal oxidant.

**Scheme 6:**
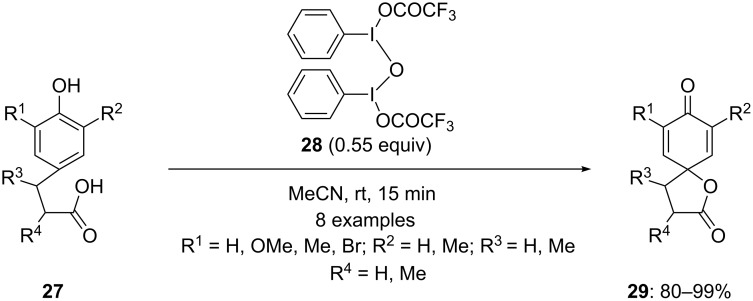
Bridged iodine(III)-mediated spirocyclization of phenols **27** to spirodienones **29**.

In 2011, Kita and co-workers [72] investigated a more reactive µ-oxo bridged hypervalent iodine(III) reagent used in the spirocyclization of phenolic substrates **27** to spirolactones **29**. The reaction products were obtained in excellent yields using 0.55 equivalents of bridged iodine(III) reagent **28** in acetonitrile at room temperature (Scheme 6). Furthermore, a comparative study was done between bridged iodine(III) reagent **28** with PIFA. It was found that the reaction products **29** were obtained in higher yield using the bridged iodine(III) reagent compared to that using PIFA. Probably, the iodine-OCOCF_3_ bond of the bridged compound **28** has a significant ionic character as the iodine–oxygen bond distance is larger than in PIFA which intends to make it more reactive than PIFA.

PIFA (**31**) is a more electrophilic iodine(III) reagent than PIDA (**15**) due to the presence of two trifluoroacetoxy groups. There are some approaches for the synthesis of spirocyclic compounds where PIFA (**31**) is used as electrophile.

**Scheme 7:**
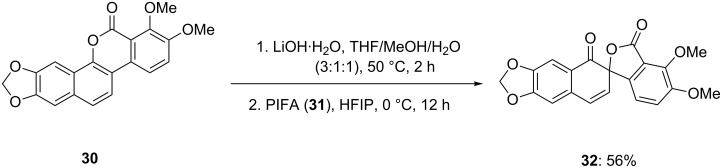
Iodine(III)-mediated spirocyclization of arnottin I (**30**) to its spirocyclic analogue arnottin II (**32**) using stoichiometric amount of PIFA (**31**).

Recently, Lewis and co-workers [73] reported the conversion of arnottin I (**30**) to its spirocyclic analogue arnottin II (**32**) by reaction with LiOH followed by PIFA (**31**). The spirocyclic product arnottin II (**32**) was isolated in 56% yield (Scheme 7). This approach is based on a tandem oxidative dearomatization process and will be quite useful for the conversion of functionalized benzocoumarins to spirocyclic lactones.

In 2015, Du and co-workers [74] reported a spirocyclization of diarylacetylenes to fused spiro polycyclic compounds through a hypervalent iodine-mediated cascade annulation reaction. In this reaction, the Lewis acid BF_3_·Et_2_O acts as catalyst which activates the substrate. A further treatment with PIDA **(15**) forms the spirocyclic products through intramolecular cyclization.

#### 2.2. Using hypervalent iodine reagents as catalyst

The hypervalent iodine-catalyzed synthesis of spirocylic compounds can be achieved either by using catalytic amounts of a hypervalent iodine species or by generation of a similar active catalytic species in situ by the oxidation of iodoarene using a terminal oxidant. More commonly, *m-*chloroperbenzoic acid (*m*CPBA) and oxone are used as oxidant to generate the hypervalent iodine species in situ via oxidation of iodoarenes. In 2014, Singh and Wirth have compiled a review article where they have covered various aspects of hypervalent iodine catalyzed reactions [75].

**Scheme 8:**
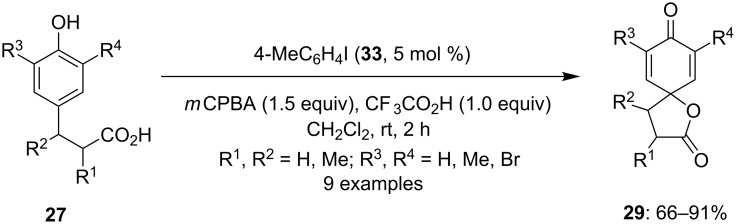
Iodine(III)-catalyzed spirolactonization of *p*-substituted phenols **27** to spirolactones **29** using iodotoluene **33** as a precatalyst and *m*CPBA as an oxidant.

In 2005, Kita and his research group investigated a hypervalent iodine-catalyzed spirocyclization reaction by generating the catalytic hypervalent iodine species via in situ oxidation of iodoarene using *m*CPBA as terminal oxidant [76]. In this report, *p*-substituted phenols **27** were cyclized to the corresponding spirolactones **29** using iodotoluene **33** as precatalyst, *m*CPBA as oxidant and TFA as an additive. The spirolactones **29** were isolated as reaction products in excellent yields (Scheme 8). Probably, the iodine(III) species was generated in situ as the active catalytic species that was playing the key role for the dearomatization of phenol. In addition, a similar reaction was also achieved by using various PIFA analogues as catalyst directly in the presence of 1.5 equivalents of *m*CPBA. Since this report, several iodine(III)-catalyzed oxidative spirocyclization reactions have been successfully developed.

**Scheme 9:**
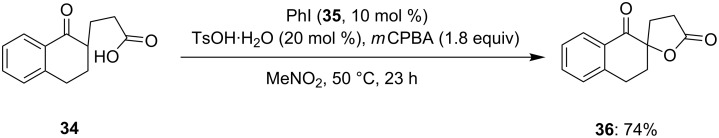
Iodine(III)-catalyzed oxylactonization of ketocarboxylic acid **34** to spirolactone **36** using iodobenzene **35** as precatalyst in the presence of *m*CPBA.

In 2009, Ishihara and co-workers [77] developed an oxylactonization of ketocarboxylic acid **34** to spirolactone **36** using 10 mol % of iodobenzene (**35**) as precatalyst, 20 mol % of TsOH·H_2_O as additive and 1.8 equivalents of *m*CPBA as oxidant. The catalytic reaction was carried out in nitromethane at 50 °C for 23 h and spirolactone **36** was isolated in 74% yield (Scheme 9). It was noted that 20 mol % of additive was essential to initiate the reaction efficiently. The reaction was quite slow when 10 mol % of additive was used. Once again, iodine(III) species was generated in situ which was probably working as active catalytic species.

#### 2.3. Stereoselective synthesis of spirolactones

**Scheme 10:**
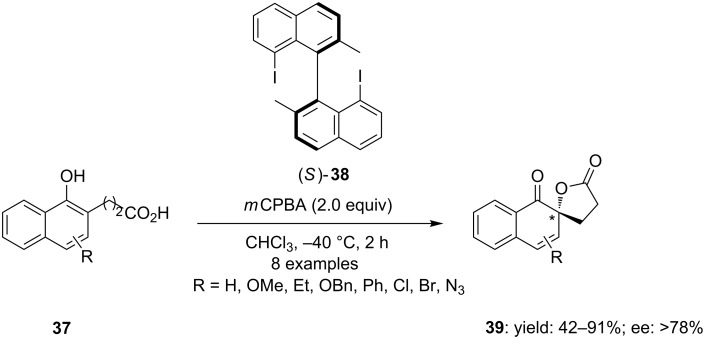
Iodine(III)-mediated asymmetric oxidative spirocyclization of naphthyl acids **37** to naphthyl spirolactones **39** using chiral iodoarene **38** as precatalyst.

Recently, Kita and co-workers [78] reported a new type of binaphthyl-based chiral iodine(III) species **38** and its efficient utilization in the spirocyclization of naphthols containing carboxylic acids. 1-Naphthol-2-propionic acids **37** were cyclized to corresponding spirolactone derivatives **39** using chiral-8,8’-diiodonaphthyl reagent **38** as precatalyst, *m*CPBA as an oxidant in chloroform at low temperature. The reaction products **39** were isolated in good yields with more than 78% enantiomeric excess (Scheme 10). The active catalytic hypervalent iodine species was generated in situ by oxidation of optically active iodoarene **38** using *m*CPBA as an oxidant.

#### 2.4. Application of spirolactones in natural products synthesis

**Scheme 11:**
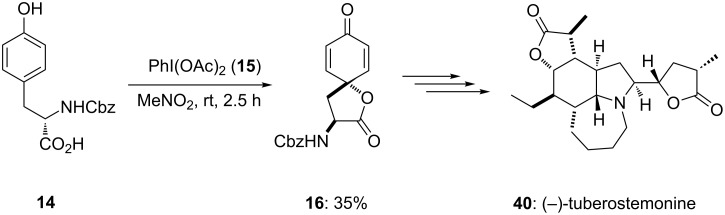
Oxidative cyclization of L-tyrosine **14** to spirocyclic lactone **16** using PIDA (**15**).

In 2005, Wipf and Spencer [79] reported the first total synthesis of the *Stemona* alkaloid (−)-tuberostemonine (**40**). In this report, PIDA (**15**) was used as an electrophile for the synthesis of spirolactone **16** in 35% yield by the cyclization of L-tyrosine **14** in nitromethane at room temperature for 2.5 h (Scheme 11). Additionally, the synthesized spirocyclic precursor **16** was transfered to (−)-tuberostemonine (**40**) in three chemical steps.

### 3. Synthesis of spirolactams

#### 3.1. Using stoichiometric amounts of iodine(III) reagents

**Scheme 12:**
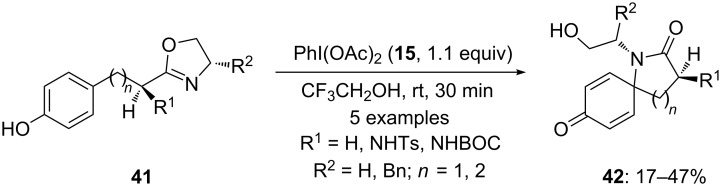
Oxidative cyclization of oxazoline derivatives **41** to spirolactams **42** using PIDA (**15**).

In 1998, Ciufolini and co-workers [80] reported the oxidative cyclization of tyrosine derivatives to spirolactams using iodine(III) reagents. In this reaction, oxazoline derivatives **41** were cyclized to spirocyclic products **42** using PIDA (**15**) as an electrophile in trifluoroethanol at room temperature for 30 minutes. The desired products **42** were isolated in moderate yields (Scheme 12).

**Scheme 13:**
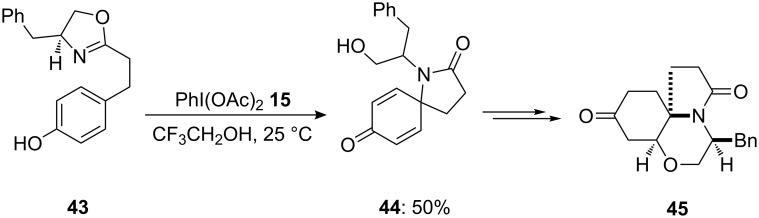
Oxidative cyclization of oxazoline **43** to spirolactam **44** using PIDA **15** as oxidant.

Additionally, the same research group [81] reported the oxidative cyclization of a phenolic substrate to a spirolactam using PIDA as electrophile. In this methodology, oxazoline **43** was cyclized to spirolactam **44** in 50% yield using PIDA (**15**) in trifluoroethanol at room temperature (Scheme 13). Furthermore, spirolactam was used as intermediate in the synthesis of tricyclic compound **43** possessing a similar structure like that of the naturally occurring heterocyclic compound FR901483 [82].

**Scheme 14:**
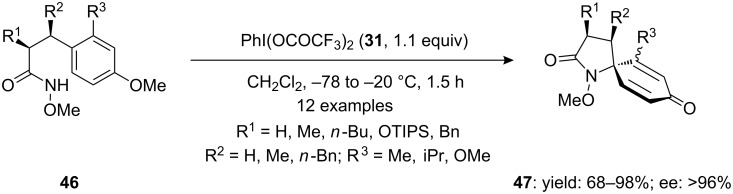
PIFA-mediated spirocyclization of amides **46** to *N*-spirolactams **47** using PIFA (**31**) as an electrophile.

Wardrop and co-workers [83] developed a new method for the preparation of 1-azaspiranes **47** by treatment of α- and β-substituted 3-(methoxyphenyl)-*N*-methoxypropionamides **46** with [bis(trifluoroacetoxy)iodo]benzene (PIFA, **31**) in dichloromethane (Scheme 14). The reactions were carried out at low temperature and spirolactams **47** were achieved in high yields with up to 96% enantiomeric excess. Furthermore, these compounds have been employed as important synthetic intermediates for the construction of biologically active molecules such as histrionicotoxins and the cytotoxic marine alkaloid fasicularin [84].

**Scheme 15:**
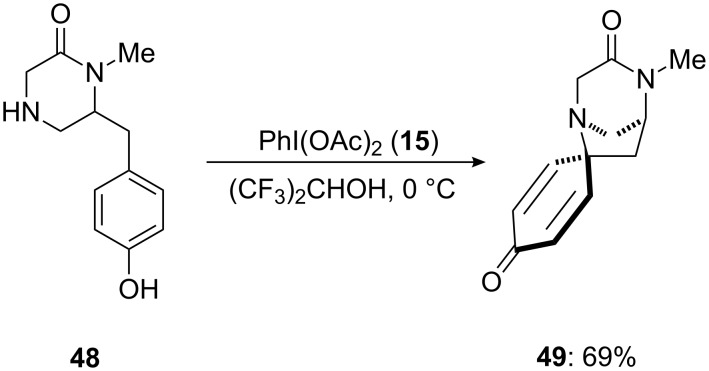
Synthesis of spirolactam **49** from phenolic enamide **48** using PIDA (**15**).

In 2010, Honda [85] reported the synthesis of isoquinoline alkaloids possessing spirocyclic framework using PIDA (**15**) as an electrophile in hexafluroisopropanol solvent. The *p*-substituted phenolic compound **48** was used as starting material for the construction of spirolactam **49** in 69% yield (Scheme 15). This is an important intermediate in the synthesis of various naturally occurring alkaloids such as TAN1251A, TAN1251C and TAN1251D [86].

Wardrop and Burge [87] reported a iodine(III)-mediated oxidative spirocyclization of hydroxamates **50**. The azaspirans **51** containing quaternary carbon centers were synthesized in good to excellent yields on treating substrates **50** with PIFA (**31**) in dichloromethane/methanol (1:1, Scheme 16). The reaction products (spirolactams **51**) were obtained as inseparable mixture of *anti*- and *syn*-diastereomers.

**Scheme 16:**
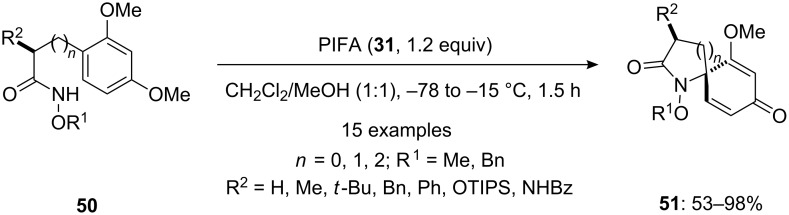
Iodine(III)-mediated spirocyclization of alkyl hydroxamates **50** to spirolactams **51** using stoichiometric amount of PIFA (**31**).

**Scheme 17:**
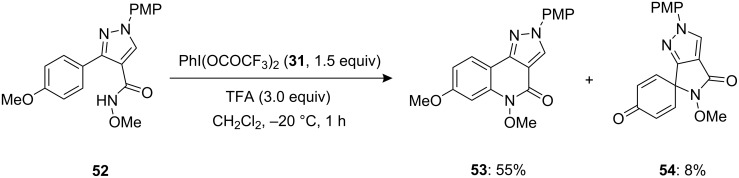
PIFA-mediated cyclization of substrate **52** to spirocyclic product **54**.

Haroutounian and co-workers [88] investigated a PIFA-mediated synthesis of spirocyclic lactam **54** as side product by treating substrate **52** with 1.5 equivalents of PIFA (**31**) in presence of 3.0 equivalents of TFA as an additive in dichloromethane (Scheme 17). The fused tricyclic compound **53** was obtained as major product in 55% yield along with the spiro compound **54** as a minor product in 8% yield.

**Scheme 18:**
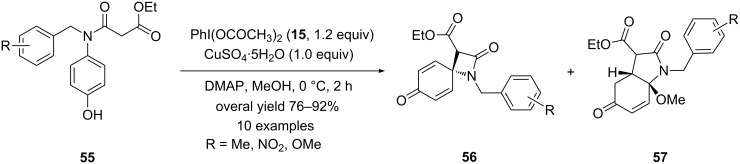
Synthesis of spiro β-lactams **56** by oxidative coupling reaction of *p*-substituted phenols **55** using PIDA (**15**) and CuSO_4_·5H_2_O in the presence of base in methanol.

In 2009, Zhang and co-workers [89] reported an efficient method for the synthesis of spiro β-lactams via oxidative dearomatization reactions. In this report, the synthesis of spiro β-lactams **56** were achieved successfully by the oxidative cyclization of *p*-substituted phenols **55** using PIDA (**15**) as an electrophile and copper(II) sulfate pentahydrate as an additive in the presence of DMAP base. The spirocyclization reactions were performed in MeOH for 2 h at 0 °C and spirocyclic products **56** were isolated in good yields (Scheme 18). Additionally, fused bicyclic compounds **57** were also observed in few reactions in traces. The structure of the spiro β-lactam was confirmed by single crystal X-ray crystallography.

Dong and co-workers [90] developed a novel way for the synthesis of five membered spiro pyrazolin-5-ones using amide and amine-containing precursors. Herein, five-membered azaheterocyclic derivatives were synthesized efficiently in presence of PIFA and with TFA as an additive.

**Scheme 19:**
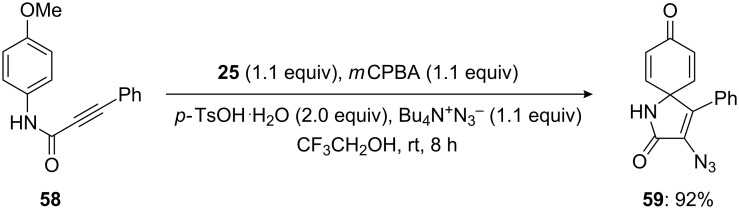
Iodine(III)-mediated spirocyclization of *para*-substituted amide **58** to spirolactam **59** by the reaction with bis(iodoarene) **25** in the presence of *m*CPBA.

Furthermore, Kita and his research group [71] displayed a method for the cyclization of alkyne derivative **58** to spirolactam **59** by an in situ-generated active hypervalent iodine species. In this method, *para*-substituted amide **58** was cyclized to the corresponding spirolactam **59** in 92% yield using a stoichiometric amount of bis(iodoarene) **25** with the terminal oxidant *m*CPBA in the presence of TsOH·H_2_O in TFE (Scheme 19).

**Scheme 20:**
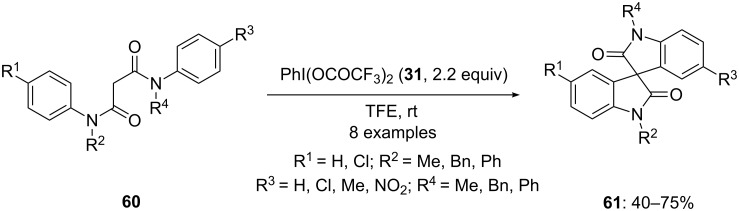
Iodine(III)-mediated synthesis of spirolactams **61** from anilide derivatives **60**.

In 2012, Zhao and co-workers [91] developed a new approach for the construction of spirooxindoles **61** through tandem cascade oxidation of substituted anilides **60**. In this methodology, anilide derivatives **60** were reacted with [bis(trifluoroacetoxy)iodo]benzene (**31**, PIFA) in TFE at room temperature to afford functionalized lactams **61** in good yields (Scheme 20). Various electron-donating and withdrawing groups at the phenyl ring in anilides were successfully tolerated.

**Scheme 21:**
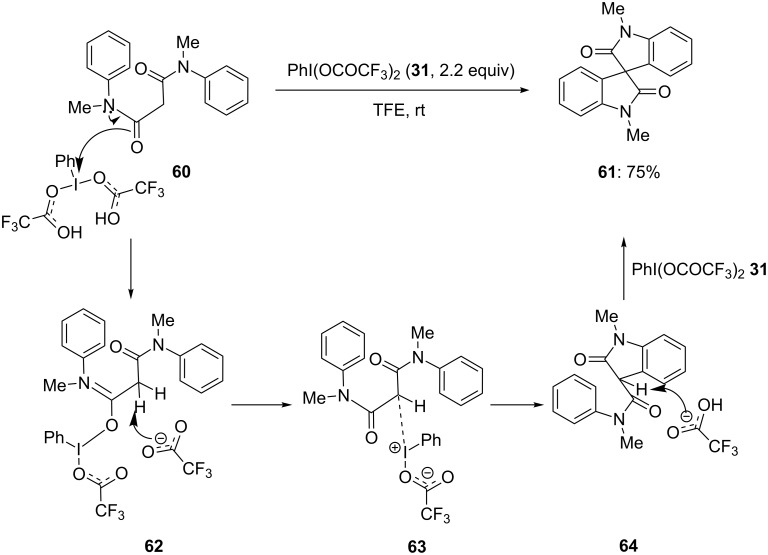
PIFA-mediated oxidative cyclization of anilide **60** to bis-spirobisoxindole **61**.

Furthermore, Sunoj and Sreenithya [92] developed a metal-free approach for the synthesis of 1,1'-dimethyl-3,3'-spirobi[indoline]-2,2'-dione (**61**) from *N*^1^,*N*^3^-dimethyl-*N*^1^,*N*^3^-diphenylmalonamide (**60**) using PIFA (**31**) in trifluoroethanol at room temperature. The spirolactam **61** was isolated in 75% yield (Scheme 21). According to the proposed mechanistic pathway, the reaction was initiated with formation of an intermediate **63** by the attack of the carbonyl oxygen to electrophilic iodine(III) reagent **31** which could be rearranged to compound **64**. Finally, the acetate anion attacks the β-hydrogen of **64** to form spirolactam product **61**.

**Scheme 22:**
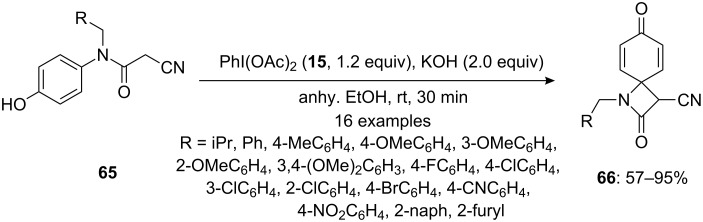
PIDA-mediated spirocyclization of phenylacetamides **65** to spirocyclic lactams **66**.

In 2014, Xu and Abdellaoui [93] reported a nucleophilic intramolecular cyclization of phenylacetamides **65** to spirocyclic lactams **66** via iodine(III)-mediated spirocarbocyclizations. In literature, there are limited methods available for the synthesis of spiro-β-lactam-3-carbonitrile which is widely used as an antibiotic [94]. In this methodology, *N*-(*p*-hydroxyphenyl)cyanoacetamides **65** were cyclized to corresponding 4-spiro-β-lactam-3-carbonitriles **66** in useful yields using PIDA (**15**) as an electrophile in the presence of KOH as base in anhydrous ethanol at room temperature (Scheme 22).

**Scheme 23:**
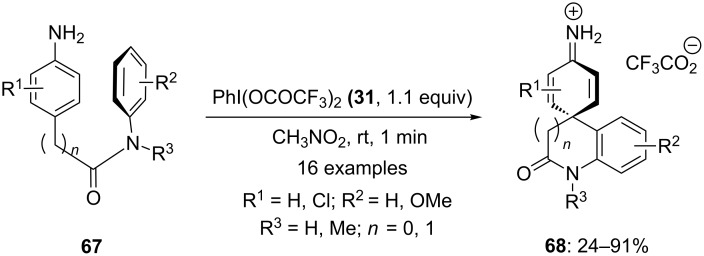
Oxidative dearomatization of arylamines **67** with PIFA (**31**) to give dieniminium salts **68**.

In 2014, Fan and co-workers [95] investigated an efficient approach for the synthesis of a spirocyclic-skeleton-containing dieniminium moiety. Herein, arylamines **67** were cyclized to spirocyclic dieniminium salts **68** using PIFA (**31**) as an electrophilic species in nitromethane (Scheme 23). All the reactions were completed within a minute and desired lactams were isolated in good yields. The presence of electron-withdrawing groups at the aromatic ring shows a negative effect on the yield while the presence of electron-enriched groups afforded the products **68** in high yields.

**Scheme 24:**
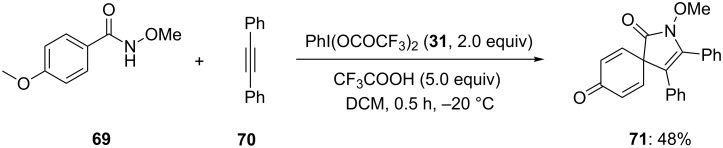
PIFA-mediated oxidative spirocarbocyclization of 4-methoxybenzamide **69** with diphenylacetylene (**70**) to spirolactam **71**.

In addition, Zhu and co-workers [96] developed another hypervalent iodine-mediated intermolecular spirocarbocyclization approach for synthesis of spirolactam. In this approach, *N*-methoxybenzamide **69** and diphenylacetylene (**70**) were treated in presence of PIFA (**31**) in dichloromethane to corresponding spirodienone compound **71** in 48% yield (Scheme 24). Additionally, trifluoroacetic acid (TFA) was used as an additive in the reaction.

**Scheme 25:**
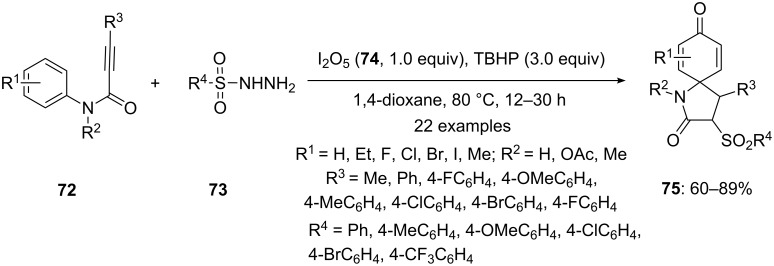
Synthesis of spiroxyindole **75** using I_2_O_5_/TBHP oxidative system.

In 2015, Wang’s group [97] reported an iodine(III)-mediated approach for the intermolecular spirocyclization of amides **72** with sulfonylhydrazides **73** to spirolactams **75**. In this method, functionalized amides **72** containing an alkyne moiety and sulfonylhydrazides **73** undergo intermolecular spirocyclization in presence of I_2_O_5_/TBHP oxidative system to give the sulfonated spirolactams **75** in high yields (Scheme 25). This oxidative system found to be more efficient and could sustain the presence of diverse functional groups. The structure of **75** was confirmed by single crystal X-ray crystallography.

#### 3.2. Using hypervalent iodine reagents as catalysts

**Scheme 26:**
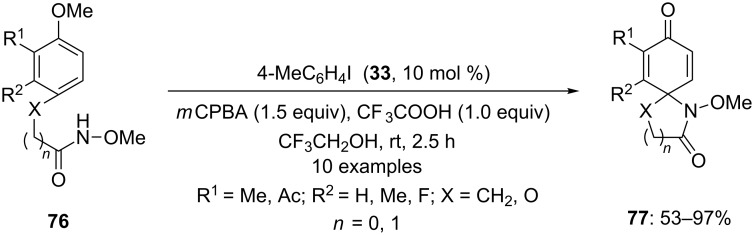
Iodine(III)-catalyzed spirolactonization of functionalized amides **76** to spirolactones **77** using iodotoluene **33** as a precatalyst and *m*CPBA as an oxidant.

In 2007, Kita and co-workers [98] investigated the first iodoarene-catalyzed spirocyclization of functionalized amides **76** to spirocyclic systems **77** by carbon–nitrogen bond formation using 10 mol % of iodotoluene **33** as precatalyst, 1.0 equivalent of CF_3_COOH as an additive and *m*CPBA as terminal oxidant in trifluoroethanol (Scheme 26). The spirocyclic compounds **77** were isolated in high yields. The cyclization reaction was probably initiated by in situ generated active iodine(III) species by the oxidation of iodotoluene **33** in the presence of *m*CPBA.

**Scheme 27:**
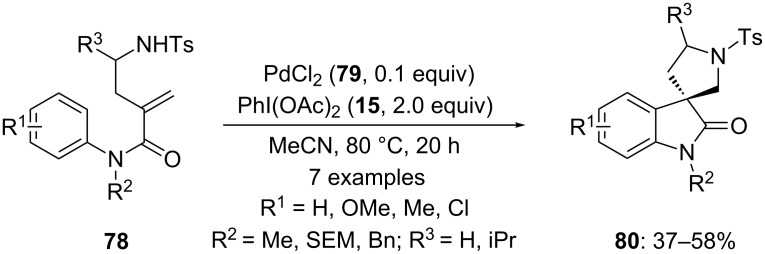
Intramolecular cyclization of alkenes **78** to spirolactams **80** using Pd(II) **79** and PIDA (**15**) as the oxidative system in acetonitrile.

In 2010, Zhu’s research group [99] achieved a Pd-catalyzed synthesis of spirolactams **80** by the cyclization of functionalized amides **78** using 10 mol % PdCl_2_ (**79**) in presence of PhI(OAc)_2_ (**15**) in acetonitrile solvent at 80 °C. The spirocyclic products **80** were obtained in moderate yields (Scheme 27). It was observed that the introduction of electron-donating group at *para*-position in substrates **78** gave the desired products in good yields whereas introduction of strong electron withdrawing groups resulted in a decrease in the yield of spirocyclic products.

**Scheme 28:**
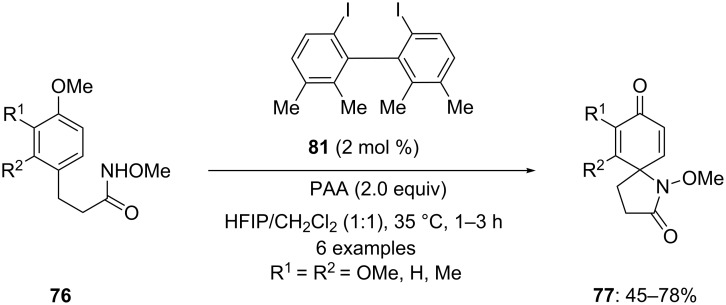
Iodine(III)-catalyzed spiroaminocyclization of amides **76** to spirolactam **77** using bis(iodoarene) **81** as a precatalyst in the presence of PAA.

Kita and co-workers [100] developed another catalytic approach for the cyclization of amides **76** to spirolactams **77**. In this approach, 2 mol % of bis(iodoarene) **81** was used as precatalyst and peracetic acid (PAA) as an oxidant instead of *m*CPBA, which plays an important role in generation of active iodine(III) species. The bis(iodoarene) **81** was oxidized to a unique µ-oxo-bridged hypervalent iodine(III) species in situ, wherein PAA is used as extremely green oxidant which releases non-toxic co-products (Scheme 28).

**Scheme 29:**
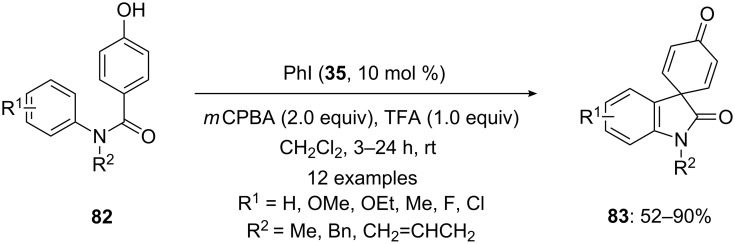
Iodine(III)-catalyzed spirolactonization of *N*-phenyl benzamides **82** to spirolactams **83** using iodobenzene **35** as a precatalyst.

In 2011, Yu and co-workers [101] developed an intramolecular lactonization of *p*-substituted phenols **82** to spirooxindoles **83** using 10 mol % of iodobenzene (**35**) as precatalyst, *m*CPBA as an external oxidant and TFA as additive. All the catalytic reactions were performed in dichloromethane and spirolactams **83** were isolated in good to excellent yields (Scheme 29). It was noted that *m*CPBA/TFA combination did not work well for some transformations and it was replaced with oxidant urea·H_2_O_2_ and TFAA as an additive.

#### 3.3. Stereoselective synthesis of spirolactams

**Scheme 30:**
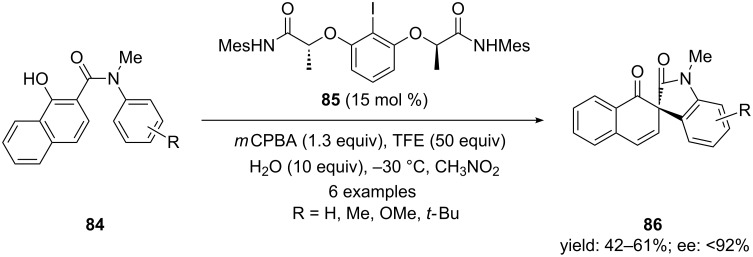
Iodine(III)-mediated asymmetric oxidative spirocyclization of phenols **84** to spirolactams **86** using chiral iodoarene **85** as precatalyst.

Gong and co-workers [102] efficiently cyclized 1-hydroxy-*N*-aryl-2-naphthamides **84** to corresponding spirolactam derivatives **86** using chiral iodoarene **85** as precatalyst, *m*CPBA as an oxidant and TFE as an additive. The presence of 10.0 equivalents of H_2_O was required to get the reaction products in high yields with up to 92% ee (Scheme 30). The chiral hypervalent-λ^3^-iodanes were generated in situ by the oxidation of the chiral *C*_2_-symmetric iodoarene **85** that was playing the key role for the oxidative spirocyclization of phenols.

**Scheme 31:**
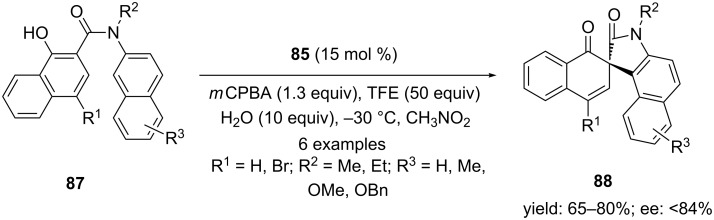
Iodine(III)-catalyzed asymmetric oxidative spirocyclization of *N*-aryl naphthamides **87** to spirocyclic compounds **88** using chiral iodoarene **85** as precatalyst.

In addition, *N*-methyl-*N*-(2-naphthyl)-2-naphthamides **87** were also cyclized to corresponding spiro compounds **88** in high yields and with upto 84% enantiomeric excess (Scheme 31). Furthermore, the absolute configuration of **88** was assigned by its single crystal X-ray analysis.

#### 3.3. Application of spirolactams in natural product synthesis

**Scheme 32:**
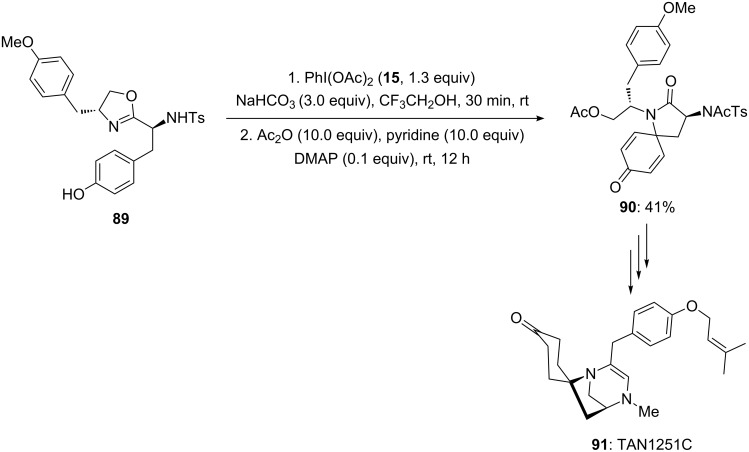
Cyclization of *p*-substituted phenolic compound **89** to spirolactam **90** using PIDA (**15**) in TFE.

In 2001, Ciufolini and co-workers [103] employed PIDA (**15**) as an electrophile during the synthesis of naturally occurring tricyclic azaspirane derivative TAN1251C. In this report, phenolic 3-arylpropionamide **89** was cyclized to spirolactam **90** in 41% yield using PIDA (**15**) as an electrophile in the presence of NaHCO_3_ in trifluoroethanol (TFE) at room temperature followed by addition of acetic anhydride and pyridine in the presence of 10 mol % DMAP (Scheme 32). In addition, spirocyclic product **90** was used as key precursor in the synthesis of naturally occurring tricyclic azaspirane derivative TAN1251C **91** in a sequence of steps.

**Scheme 33:**
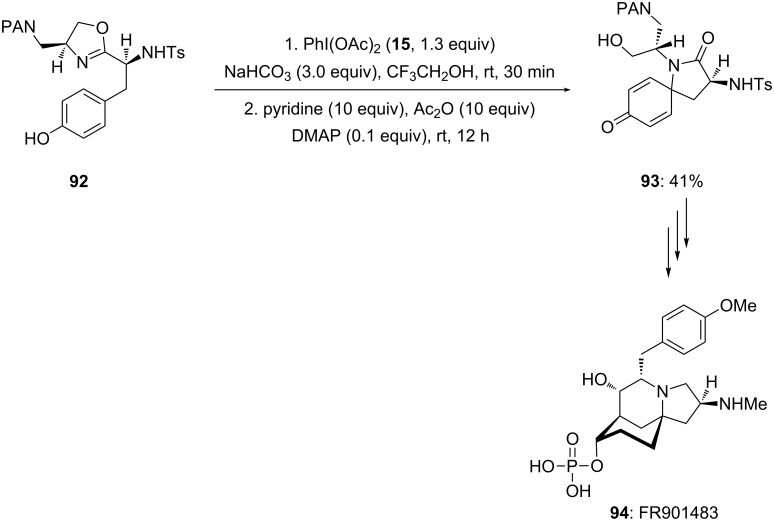
Iodine(III)-mediated synthesis of spirocyclic compound **93** from substrates **92** using PIDA (**15**) as an electrophile.

Furthermore, PIDA (**15**) was used as an electrophile during the synthesis of biologically active molecule FR901483 by the same research group [104]. In this report, spirocyclic oxazoline **93** was prepared by starting from *para*-substituted phenolic compound **92** under the reaction conditions mentioned in Scheme 32 (Scheme 33).

**Scheme 34:**
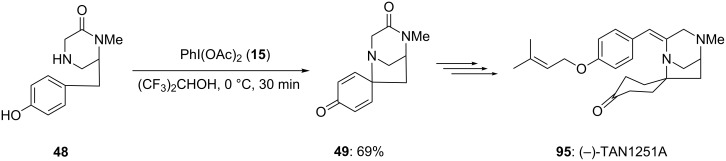
Iodine(III)-mediated spirocyclization of *p*-substituted phenol **48** to spirocyclic compound **49** using PIDA (**15**) as an electrophile.

In 2002, Honda and co-workers [105] reported the synthesis of naturally occurring (−)-TAN1251A (**95**) employing an oxidation of phenols via an dearomatization process. In this report, *para*-substituted phenolic compound **48** was cyclized to spirocyclic lactam **49** using PIDA (**15**) as an electrophile. The spirocyclic compound **49** was achieved in 69% yield (Scheme 34). Additionally, synthesized spirocyclic compound **49** was converted to natural product **95** in few chemical steps.

### 4. Synthesis of spirocarbocycles

#### 4.1. Using stoichiometric amounts of iodine(III) reagents

**Scheme 35:**
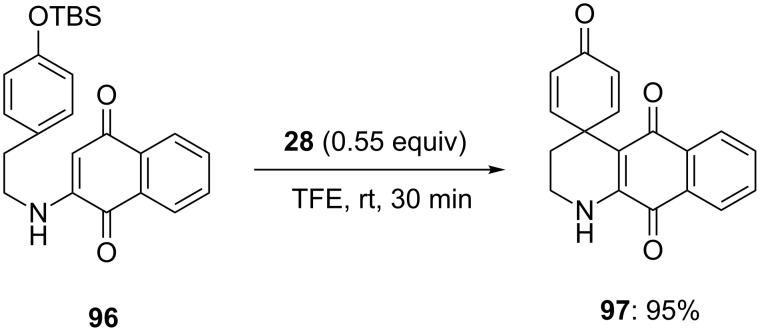
Bridged iodine(III)-mediated spirocyclization of *O*-silylated phenolic compound **96** in the synthesis of spirodienone **97**.

Furthermore, *O*-silylated phenolic compound **96** was spirocyclized to spirocarbocyclic compound **97** in 95% yield using bridged iodine(III) reagent **28** as an electrophile and trifluoroethanol (TFE) as the solvent at room temperature (Scheme 35). Compound **97** was further used as substrate for the synthesis of discorhabdin alkaloids [106–107].

**Scheme 36:**
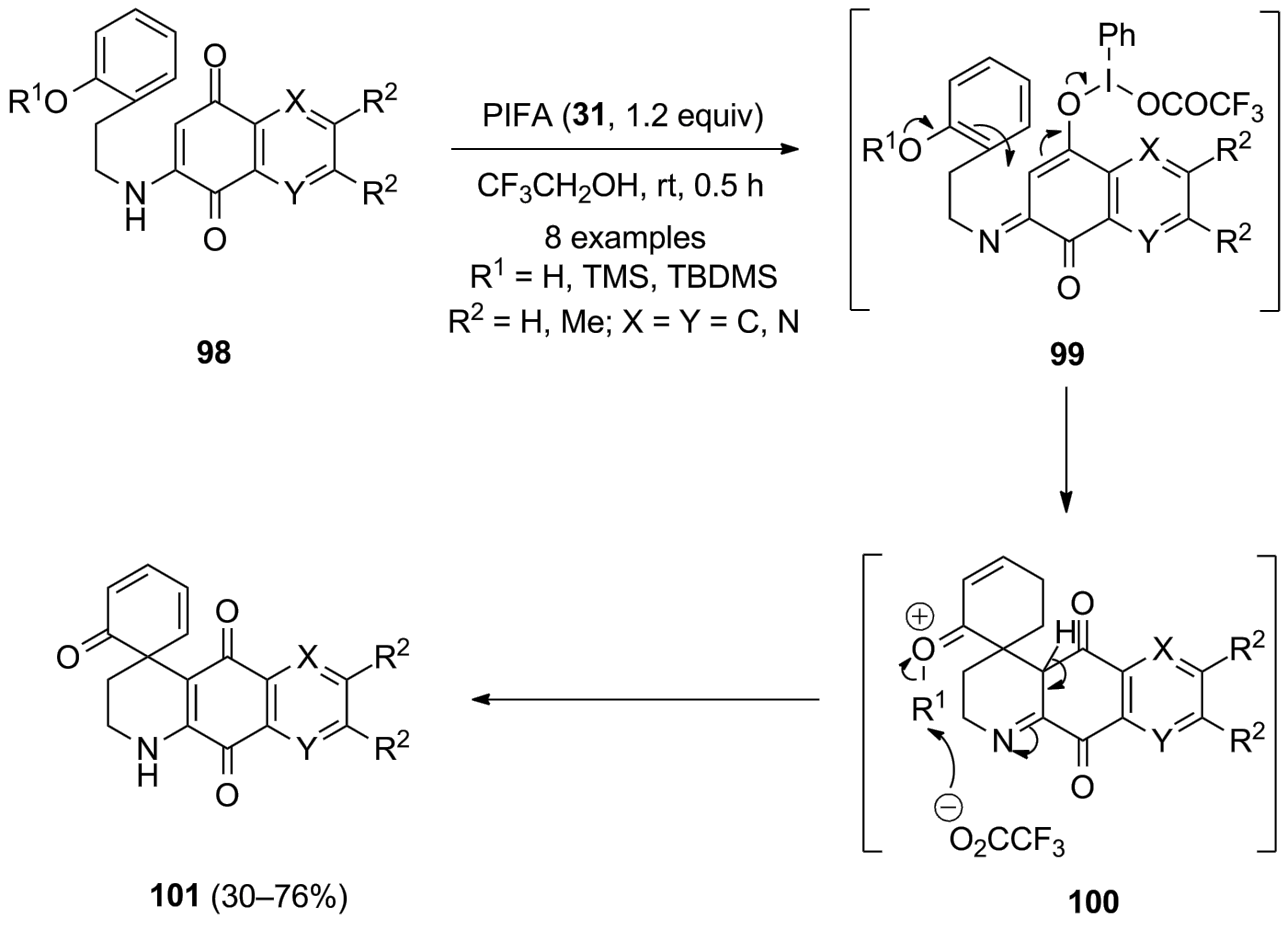
PIFA-mediated approach for the spirocyclization of *ortho-*substituted phenols **98** to aza-spirocarbocyclic products **101**.

In 1996, Kita and co-workers [108] developed an intramolecular cyclization of *ortho-*substituted phenols **98** to aza-spirocarbocyclic compounds **101** via hypervalent iodine-mediated spirocarbocyclization reactions using **31** as an electrophile. In this methodology, *ortho*-substituted phenolic derivatives **98** were treated with stoichiometric amounts of PIFA (**31**) in trifluoroethanol at room temperature for 0.5 h to afford spirocyclic compounds **101** in good yields (Scheme 36).

**Scheme 37:**
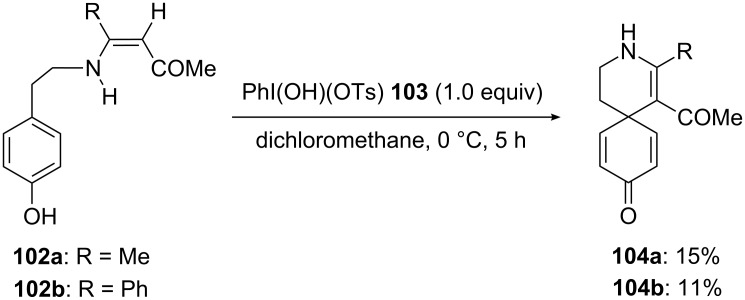
Oxidative cyclization of *para*-substituted phenols **102** to spirocarbocyclic compounds **104** using Koser reagent **103**.

Like PIDA and PIFA, Koser reagents are other iodine(III) reagents known to behave as electrophiles. In 2000, Spyroudis and co-workers [109] reported the spirocyclization of *para*-substituted phenols **102** to corresponding spirocarbocyclic derivatives **104** via dearomatization process using Koser reagent. In this reaction, substrates **102** were reacted with a stoichiometric amount of [(hydroxy)(tosyloxy)iodo]benzene (**103**) in dichloromethane at 0 °C. The spirocyclic products **104** were obtained in poor yields (Scheme 37).

**Scheme 38:**
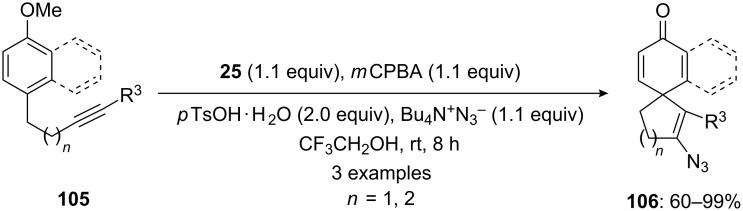
Iodine(III)-mediated spirocyclization of aryl alkynes **105** to spirocarbocyclic compound **106** by the reaction with bis(iodoarene) **25** in the presence of *m*CPBA.

Furthermore, Kita and his research group [71] reported the synthesis of spirocarbocyclic compounds **106** from arylalkynes **105** using a hypervalent iodine reagent generated in situ by the oxidation of bis(iodoarene) **25** in the presence of *m*CPBA as an terminal oxidant (Scheme 38).

**Scheme 39:**
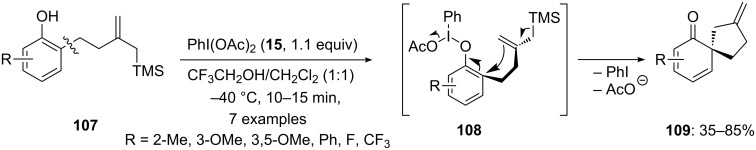
Iodine(III)-mediated spirocarbocyclization of *ortho*-substituted phenols **107** to spirocarbocyclic compounds **109** using PIDA **15**.

Wang and co-workers [110] developed a hypervalent iodine-mediated synthesis of *ortho*-spirocarbocyclic compounds via dearomatization of *ortho*-substituted phenols. In this reaction, *ortho*-substituted phenols **107** were cyclized to form spirocarbocyclic compounds **109** in useful yields. All the reactions were performed in a CF_3_CH_2_OH/CH_2_Cl_2_ (1:1) solvent combination using PIDA (**15**) as an electrophile at −40 °C for 10–15 minutes (Scheme 39). This is an example of an *ortho*-oxidative phenol dearomatization reaction wherein there is the formation of the steriogenic center at the spiro-ring junction. This approach provides an easy and direct method for the construction of *ortho*-spirocarbocyclic compounds which is broadly found to originate in most of bioactive natural products [111–112].

#### 4.3. Application of spirocarbocyclic compounds in natural product synthesis

**Scheme 40:**
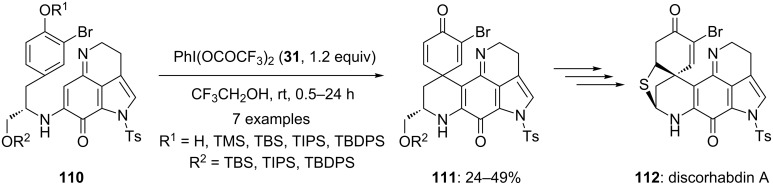
PIFA-mediated oxidative cyclization of substrates **110** to spirocarbocyclic compounds **111**.

In 2003, Kita and co-workers [113–114] employed a iodine(III) reagent during the total synthesis of sulfur-containing alkaloid **112**. Initially, the substrates **110** were cyclized to spirodienone derivatives **111** in useful yields using PIFA (**31**) as source of electrophile in trifluoroethanol at room temperature (Scheme 40). Furthermore, synthesized compounds **111** were converted into the natural product discorhabdin A (**112**).

**Scheme 41:**
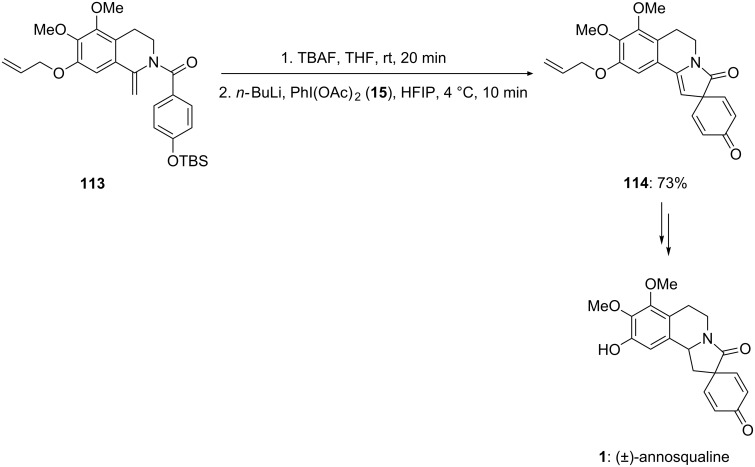
Iodine(III)-mediated cyclization of substrate **113** to spirocyclic compound **114**.

In 2006, Honda and co-workers [115] reported the total synthesis of spiro-isoquinoline alkaloid (±)-annosqualine (**1**). In this report, the substrate **113** was cyclized to form spirocyclic compound **114** via desilylation with TBAF in THF followed by reaction with *n*-BuLi in hexafluoroisopropanol using PIDA (**15**) at 4 °C (Scheme 41). This oxidative cyclization of enamide substrate **113** afforded synthetically useful spiroenamide **114**, which was used as key intermediate for total synthesis of annosqualine (**1**). The synthesis of natural product **1** was achieved in two steps starting from synthesized compound **114**.

**Scheme 42:**
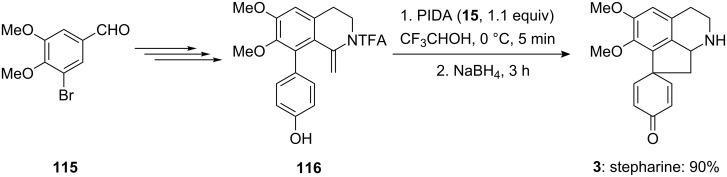
Iodine(III)-mediated spirocyclization of phenolic substrate **116** to the spirocarbocyclic natural product stepharine **3** using PIDA (**15**).

Honda and Shigehisa [116] reported the total synthesis of naturally occuring compound stepharine (**3**) starting from aromatic aldehyde **115**. Initially, substituted phenolic compound **116** was prepared in seven steps from aldehyde **115**. Furthermore, the synthesized compound **116** was converted into the natural product stepharine (**3**) by reaction with PIDA (**15**) in trifluoroethanol (TFE) followed by the reduction with NaBH_4_. The synthesis of the natural product stepharine (**3**) was obtained in 90% yield by starting from phenolic substrate **116** (Scheme 42).

**Scheme 43:**
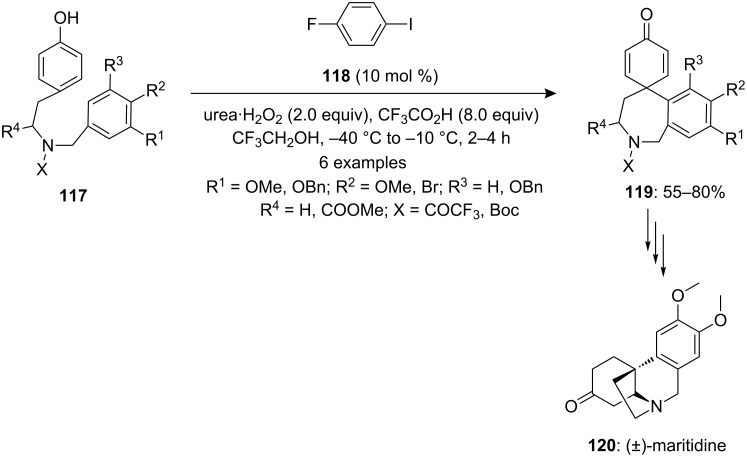
Iodine(III)-catalyzed spirocyclization of phenols **117** to spirocarbocyclic products **119** using iodoarene **118** in the presence of the oxidant urea·H_2_O_2_.

In 2008, Kita and co-workers [117] developed an iodine(III)-catalyzed approach for the spirocyclization of *p*-substituted phenols **117** to spirocarbocyclic products **119** in good yields using a catalytic amount of iodoarene **118** and urea·H_2_O_2_ as an oxidant. Probably, the active hypervalent iodine(III) species was generated in situ by the oxidation of iodoaerene **118** in the presence of urea·H_2_O_2_ oxidant (Scheme 43). Furthermore, the synthesized spirocyclic compounds were used as synthetic intermediate for the synthesis of biologically active natural product amaryllidaceae alkaloids such as (±)-maritidine (**120**) [118–120].

**Scheme 44:**
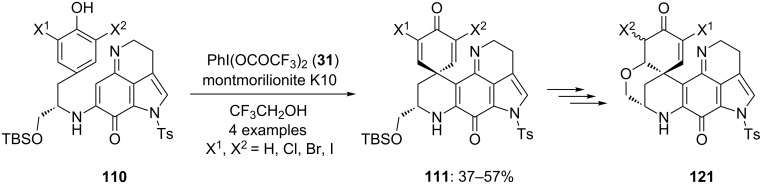
PIFA**-**mediated spirocyclization of **110** to spirocyclic compound **111** using PIFA (**31**) as electrophile.

In 2009, Kita and co-workers [121] reported the synthesis of various oxygen analogues of naturally occurring compound discorhabdin A starting from substrate **110** in few chemical steps. Discorhabdin A is an alkaloid that shows various biological activities including strong cytotoxic activity [122]. During the first step, starting substrates **110** were cyclized to spirocyclic compounds **111** in useful yields using PIFA (**31**) in presence of montmorillonite K10 in trifluoroethanol (Scheme 44). Furthermore, synthesized spirocyclic compounds **111** were used as key precursors for the synthesis of oxygen analogues of discorhabdin A (**121**).

### 5. Synthesis of miscellaneous spirocyclic compounds

#### 5.1. Using stoichiometric amounts of iodine(III) reagents

**Scheme 45:**
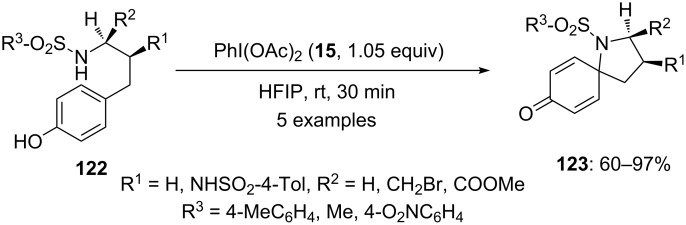
PIDA-mediated spirocyclization of phenolic sulfonamide **122** to spiroketones **123**.

In 2002, Ciufolini and co-workers [123] reported the spirocyclization of various phenolic sulfonamides **122** to spiropyrrolidines **123** using PIDA (**15**). In this reaction, sulfonamides **122** undergo N-acylation, wherein various homotyramine sulfonamides were treated with electrophile PIDA (**15**) in hexafluoroisopropanol to give the spirocyclic products **123** in high yields (Scheme 45). However, the similar spirocyclization could not successfully applied for the construction of six-membered spiropiperidine systems.

**Scheme 46:**
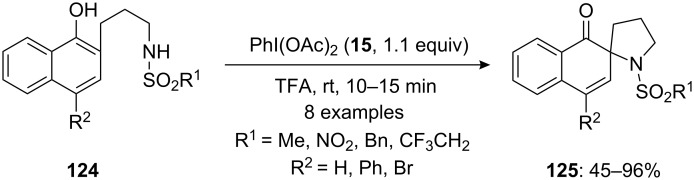
Iodine(III)-mediated oxidative spirocyclization of 2-naphthol derivatives **124** to spiropyrrolidines **125**.

In 2015, Jain and Ciufolini [124] developed PIDA-mediated spirocyclization of 2-naphtholic sulfonamides **124** to spiropyrrolidine derivatives **125**. The spirocyclization reactions were carried out by treating *N*-sulfonamide substrates **124** with (diacetoxyiodo)benzene (**15**) in trifluoroacetic acid (TFA) and spiropyrrolidines **125** were isolated in good to excellent yields (Scheme 46). However, the presence of an electron-donating functionality at *para*-position to the phenolic group induced no spirocyclization product.

**Scheme 47:**
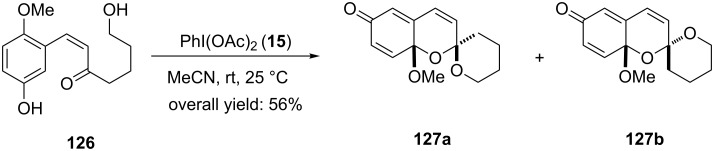
PIDA-mediated oxidative spirocyclization of *m*-substituted phenols **126** to tricyclic spiroketals **127**.

In 2016, Bray and Shirley [125] reported the oxidative spirocyclization of *meta*-substituted phenol **126** to tricyclic spiroketals **127a**,**b** in 56% yield using PIDA (**15**) as electrophilic species in acetonitrile at room temperature (Scheme 47). The mixture of both isomers was separated by flash column chromatography and the stereochemistry of major isomer **127a** was assigned on the basis of NOE. This spirocyclic functionality is the basic nucleus found in the *phorbaketal* family of natural products.

#### 5.2. Stereoselective synthesis of chiral spirocyclic ketals

**Figure 3:**
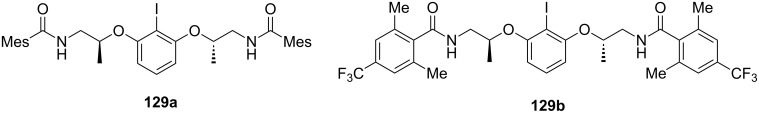
The structures of chiral organoiodine(III) catalysts **129a** and **129b**.

Recently, Ishihara and co-workers [126] synthesized chiral *C*_2_-symmetric iodoarene **129a** and **129b** (Figure 3) in few steps and used as precatalyst in iodine(III)-catalyzed enantioselective synthesis of spiroketals with high selectivities.

**Scheme 48:**
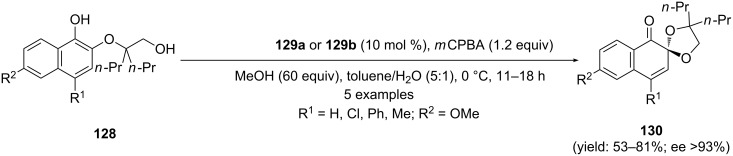
Iodine(III)-catalyzed oxidative spirocyclization of substituted phenols **128** to spirocyclic ketals **130**.

In this report, substrates **128** were reacted with 10 mol % of chiral iodoarene **129a** and **129b** in the presence of *m*CPBA oxidant in chloroform at 0 °C. The desired *ortho*-spirocyclic ketals **130** were obtained in high yields with more than 93% enantiomeric excess (Scheme 48). Interestingly, the higher selectivities were observed with chiral hypervalent iodine(III) reagent **129b** compared to **129a**.

#### 5.3. Application of miscellaneous spirocyclic compounds in natural product synthesis

**Scheme 49:**
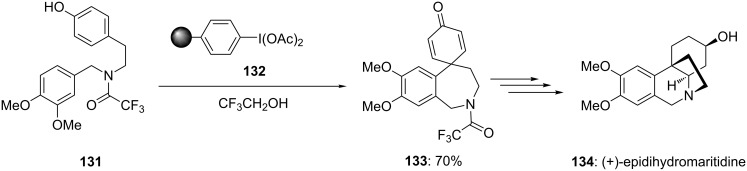
Oxidative spirocyclization of *para*-substituted phenol **131** to spirodienone **133** using polymer supported iodine(III) reagent **132**.

Various hypervalent iodine reagents have been proved as vital reagents during the synthesis of several natural products containing spirocyclic skeleton. In 1999, Ley and co-workers [127] used polymeric PIDA reagent **132** to achieve the synthesis of spirocyclic core of natural product (+)-epidihydromaritidine (**134**). In this report, *para*-substituted phenol **131** was cyclized to spirodienone **133** using polymer supported (diacetoxy)iodobenzene reagent **132** (Scheme 49). The desired product **133** was obtained in 70% yield without conventional work-up procedure and purification by chromatographic technique. Furthermore, synthesized spirocyclic compound **133** was converted into the alkaloid (+)-epidihydromaritidine (**134**) in three chemical steps.

**Scheme 50:**
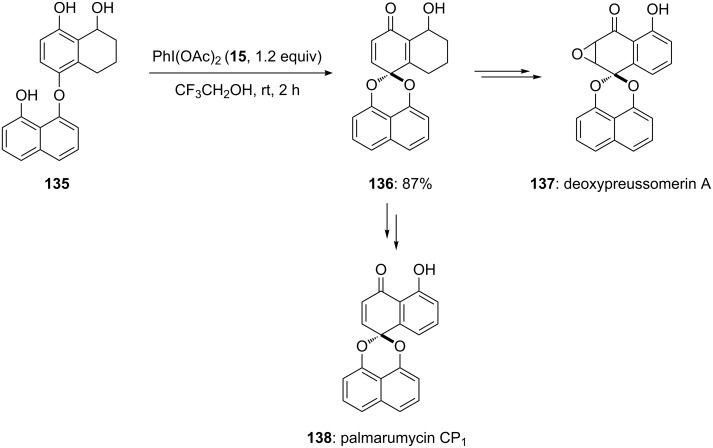
Oxidative cyclization of bis-hydroxynaphthyl ether **135** to spiroketal **136** using PIDA (**15**) as an electrophile.

Furthermore, Wipf and co-workers [128] reported a new synthetic route for the synthesis of deoxypreussomerin A **(137**) and palmarumycin CP_1_ (**138**). During the first step, the synthesis of spirocyclic compound **136** was achieved in 87% yield by the reaction of PIDA (**15**) with the naphthol derivative **135** in trifluoroethanol at room temperature. Additionally, synthesized compound **136** was used as key intermediate in the total synthesis of natural products **137** and **138** (Scheme 50). Additionally, more analogues of palmarumycin CP_1_ were synthesized later which were showing good thioredoxin–thioredoxin reductase (Trx-1/TrxR) inhibitory activity [129]. It was observed that the introduction of enone functionality in naphthoquinone spiroketal enhances the biological activity of palmarumycin **138**.

**Scheme 51:**
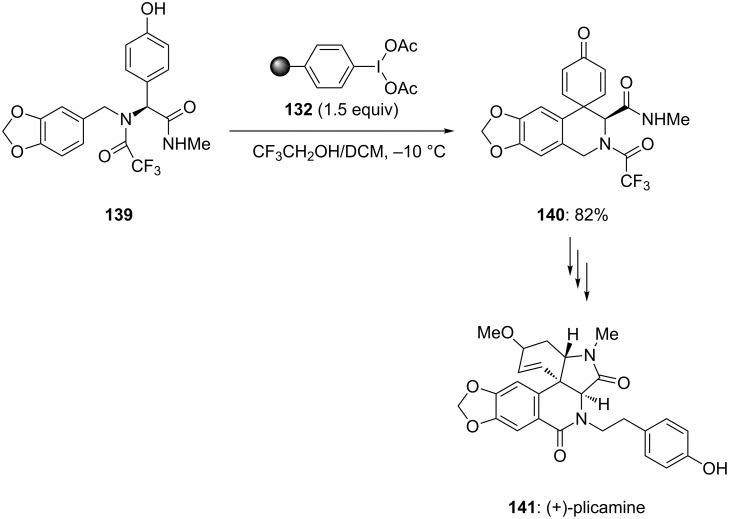
Oxidative spirocyclization of phenolic compound **139** to spirodienone **140** using polymer-supported PIDA **132**.

Furthermore, Ley and co-workers [130] employed polymer-supported iodine(III) reagent during the total synthesis of *Amaryllidaceae* alkaloid (+)-plicamine (**141**). In this report, spirodienone **140** was synthesized in 82% yield by the oxidative spirocyclization of *p*-substituted phenolic substrate **139** using polymer-supported iodonium diacetate **132** in 2,2,2-trifluoroethanol/DCM at −10 °C (Scheme 51). Additionally, the synthesized functionalized spirodienone **140** was used as precursor for the synthesis of (+)-plicamine (**141**).

**Scheme 52:**
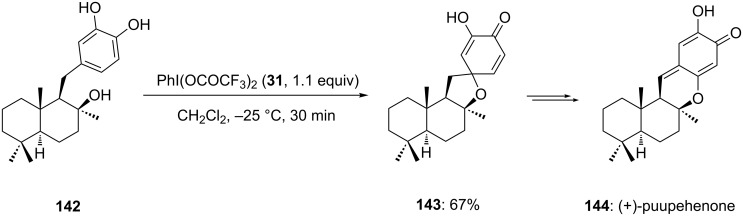
PIFA-mediated oxidative cyclization of catechol derived substrate **142** to spirocyclic product **143**.

In 2002, Quideau and co-workers [131] developed the synthesis of marine sesquiterpenoid (+)-puupehenone starting from catechol derivative **142**. Marine sesquiterpenoids are mainly known for their biological importance such as antitumor, antiviral and antibiotic properties [132]. In this report, the catechol-derived starting substrate **142** was cyclized to spirocyclic product **143** in 67% yield using PIFA (**31**) as suitable electrophile in dichloromethane at −25 °C (Scheme 52). Furthermore, the spirocyclic product **143** assists as the key synthetic intermediate in the synthesis of the marine natural product (+)-puupehenone (**144**).

**Scheme 53:**
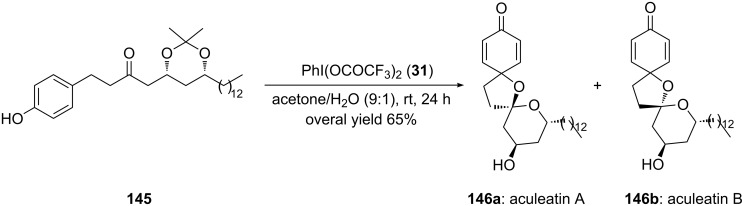
Oxidative spirocyclization of *p*-substituted phenolic substrate **145** to aculeatin A (**146a**) and aculeatin B (**146b**) using PIFA (**31**).

In 2005, Marco and co-workers [133] reported the synthesis of naturally occurring spiroacetals aculeatin A (**146a**) and aculeatin B (**146b**) and iodine(III) reagent was used as an electrophile in one step during their synthesis. In this report, *p*-substituted phenolic substrate **145** was directly cyclized to naturally occuring spirocyclic optical isomers **146a** and **146b** using PIFA (**31**) in solvent combination of CH_3_COCH_3_/H_2_O (9:1) at room temperature for 24 h. The spirocyclic compound **146** was obtained as two optical isomers in 5.5:1 ratio with overall 65% yield (Scheme 53).

**Scheme 54:**
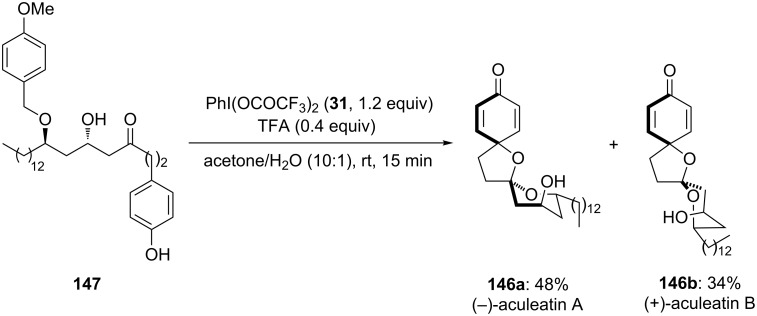
Oxidative spirocyclization of *p*-substituted phenolic substrate **147** to aculeatin A (**146a**) and aculeatin B (**146b**) using PIFA (**31**).

Furthermore, Peuchmaur and Wong [134] developed a new synthetic route for the total synthesis of the natural product (±)-aculeatin starting from substrate **147**. (±)-Aculeatin and its derivatives possessing spirocyclic skeleton are known for their antibacterial and antiprotozoal properties [135]. In this report, substrate **147** was cyclized to spiroketals, i.e., (−)-aculeatin (**146a**) and (+)-**146b** in 3:2 ratio. Herein, 1.0 equivalent of PIFA (**31**) was used as an electrophile, 0.4 equivalents of TFA as non-nucleophilic counter anion in solvent combination of Me_2_CO/H_2_O (10:1) at room temperature for 15 minutes (Scheme 54). The reaction proceeds through phenolic oxidative cyclization of phenolic substrate **147** which is the key step in the overall synthesis. The absolute configuration of the synthesised compound was determined by comparing the optical rotary values with that of natural compound (−)-aculeatin (**146a**) and (+)-aculeatin (**146b**).

**Scheme 55:**
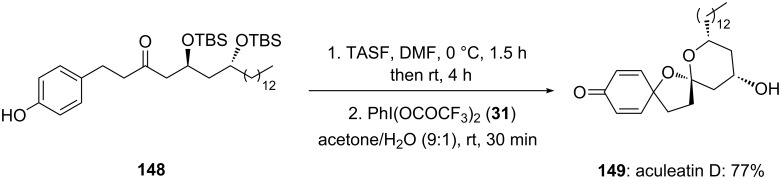
Oxidative spirocyclization of *p*-substituted phenolic substrate **148** to aculeatin D (**149**) using electrophilic species PIFA (**31**).

In the continuation to previous work, the same research group [136] reported the synthesis of aculeatin D. In this report, the *p*-substituted phenolic compound **148** was directly cyclized to natural product aculeatin D (**149**) in 77% yield using PIFA (**31**) (Scheme 55).

**Scheme 56:**
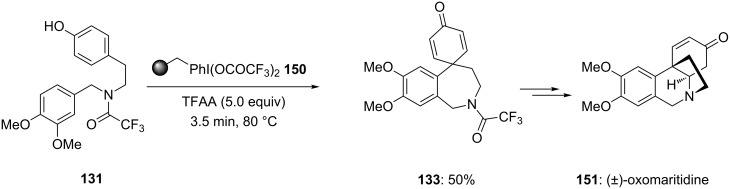
Cyclization of phenolic substrate **131** to spirocyclic product **133** using polymer-supported PIFA **150**.

In 2006, Ley and co-workers [137] reported the total synthesis of natural product (±)-oxomaritidine (**151**) starting from phenolic substrates and polymer-supported hypervalent iodine reagent was used in one step. In this report, *p*-substituted phenolic compound **131** was cyclized to spirocyclic compound **133** in 50% yield containing a seven membered ring system. The cyclization reaction was carried out using polymer-supported PIFA reagent **150** as an electrophile and trifluoroacetic anhydride (TFAA) as an additive at 80 °C in a microreactor without using any solvent (Scheme 56). Additionally, synthesised compound **133** was used as precursor for the synthesis of (±)-oxomaritidine (**151**).

**Scheme 57:**
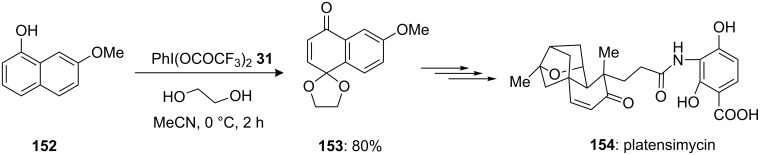
Iodine(III)-mediated oxidative intermolecular spirocyclization of 7-methoxy-α-naphthol (**152**) to spirocyclic compound **153**.

In 2007, Lalic and Corey [138] reported the synthetic pathway for the synthesis of the naturally occurring antibiotic platensimycin (**154**) which is isolated from *Streptomyces platensis*. In this report, 6-methoxy-1,4-naphthoquinone-4-ethylene ketal (**153**) was synthesized by intermolecular oxidative cyclization of 7-methoxy-α-naphthol (**152**) with ethylene glycol in the presence of PIFA (**31**) in acetonitrile. The reaction product **153** was isolated in 80% yield (Scheme 57). Additionally, the synthesised compound **153** was converted into the antibiotic platensimycin (**154**) after nine chemical steps.

**Scheme 58:**
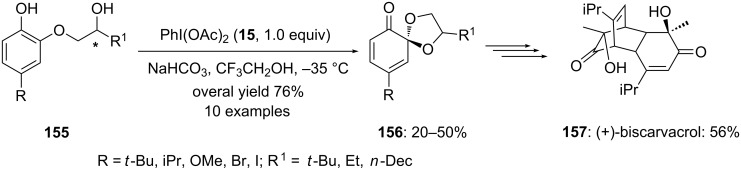
Oxidative cyclization of phenols **155** to spiro-ketals **156** using electrophilic species PIDA (**15**).

Furthermore, the same electrophilic species **15** was used to cyclize *ortho*-substituted phenolic compounds **155** to spiroketals **156** by Quideau and co-workers [139]. The cyclization reactions were performed in trifluoroethanol and spirocyclic ketals **156** were isolated in useful yields (Scheme 58). Additionally, the synthesized spiroketal **156** (R = iPr; R^1^ = iPr) was used as substrate for the synthesis of natural product (+)-biscarvacrol (**157**).

Koag and Lee [140] reported the synthesis of a spiroketal by radical cyclization of a steroidal alkylamine in presence of PIDA (**15**) as oxidant and molecular iodine in dichloromethane at low temperature. It is an example of hypoiodite-mediated radical cyclization wherein the oxazaspiroketal moiety is formed which is further used as key intermediate for the synthesis of the natural product cephalostatin.

**Scheme 59:**
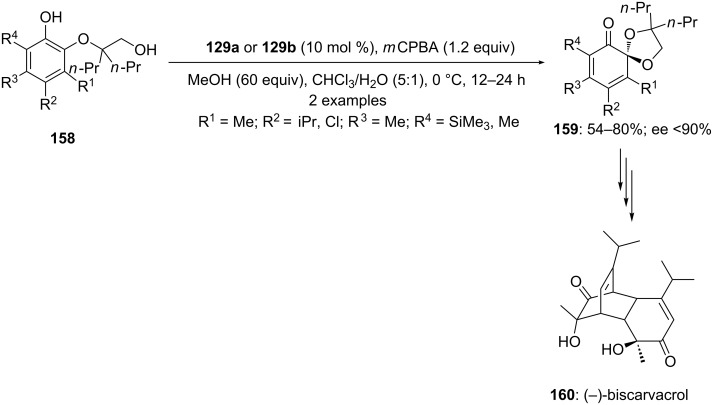
Iodine(III)-catalyzed oxidative spirocyclization of *ortho*-substituted phenols **158** to spirocyclic ketals **159**.

Additionally, spiroketals **159** were also synthesised by enatioselective spirocyclization of *ortho*-substituted phenols **158** using similar chiral auxiliaries **129a** or **129b** under similar reaction conditions mentioned in Scheme 48. Furthermore, the synthesized spiroketal **159** (R^2^ = iPr; R^4^ = SiMe_3_) was used as synthetic intermediate for enantioselective synthesis of natural product (−)-biscarvacrol [8] (Scheme 59). Additionally, Parra and Reboredo compiled a review article where authors have covered various aspects of stereoselective spirocyclizations using chiral hypervalent iodine reagents [44]. This review article would be more interesting for readers and provides some significant information about the utility of chiral iodine(III) reagents in enantioselective spirocyclizations with suitable detail.

## Conclusion

In this review article, we have summarized different approaches for the synthesis of spirocyclic scaffolds using hypervalent iodine reagents in stoichiometric or catalytic amounts. Various iodine(III) reagents such as (diacetoxyiodo)benzene, [bis(trifluoroacetoxy)iodo]benzene and Koser’s reagent have been used to achieve a variety of spirocyclization reactions under mild reaction conditions. Various hypervalent iodine-catalyzed spirocyclization of functionalized phenols and aromatic amines have been successfully developed using iodoarenes as precatalyst in the presence of terminal oxidants. In addition, this review highlights various stereoselective spirocyclizations using chiral hypervalent iodine reagents. Finally, the recent applications of hypervalent iodine reagents in natural product synthesis are also covered.

## References

[1] Nemoto T, Hamada Y (2015). Yuki Gosei Kagaku Kyokaishi.

[2] Li Y, Cheng L, Liu X, Li B, Sun N (2014). Beilstein J Org Chem.

[3] Zuo Z, Yang X, Liu J, Nan J, Bai L, Wang Y, Luan X (2015). J Org Chem.

[4] Sun W, Li G, Hong L, Wang R (2016). Org Biomol Chem.

[5] Petersen A B, Rønnest M H, Larsen T O, Clausen M H (2014). Chem Rev.

[6] Bhakuni D S, Gupta S (1982). J Nat Prod.

[7] Pouységu L, Deffieux D, Quideau S (2010). Tetrahedron.

[8] Pouységu L, Chassaing S, Dejugnac D, Lamidey A-M, Miqueu K, Sotiropoulos J-M, Quideau S (2008). Angew Chem, Int Ed.

[9] Zheng Y, Tice C M, Singh S B (2014). Bioorg Med Chem Lett.

[10] Zheng Y-J, Tice C M (2016). Expert Opin Drug Discovery.

[11] Yang Y-L, Chang F-R, Wu Y-C (2004). Helv Chim Acta.

[12] Oxford A E, Raistrick H, Simonart P (1939). Biochem J.

[13] Davenport-Hines R P T, Slinn J (1992). Glaxo: A History to 1962.

[14] Cava M P, Nomura K, Schlessinger R H, Buck K T, Douglas B, Raffauf R F, Weisbach J A (1964). Chem Ind.

[15] Ohtake Y, Sato T, Kobayashi T, Nishimoto M, Taka N, Takano K, Yamamoto K, Ohmori M, Yamaguchi M, Takami K (2012). J Med Chem.

[16] Olver I (2015). Lancet Oncol.

[17] Basarab G S, Doig P, Galullo V, Kern G, Kimzey A, Kutschke A, Newman J P, Morningstar M, Mueller J, Otterson L (2015). J Med Chem.

[18] Wirth T (2001). Angew Chem.

[19] Wirth T, Schmalz H-G, Wirth T (2003). Organic Synthesis Highlights.

[20] Wirth T, Wirth T (2003). Hypervalent Iodine Chemistry. Topics in Current Chemistry.

[21] Zhdankin V V, Stang P J (2002). Chem Rev.

[22] Wirth T (2005). Angew Chem.

[23] Zhdankin V V, Stang P J (2008). Chem Rev.

[24] Zhdankin V V (2009). ARKIVOC.

[25] Farooq U, Shah A-u-H A, Wirth T (2009). Angew Chem.

[26] Zhdankin V V (2011). J Org Chem.

[27] Singh F V, Wirth T, Molander G A, Knochel P (2014). Oxidative Functionalization with Hypervalent Halides. Comprehensive Organic Synthesis II.

[28] Moriarty R M (2005). J Org Chem.

[29] Ladziata U, Zhdankin V V (2007). Synlett.

[30] Merritt E A, Olofsson B (2009). Angew Chem.

[31] Yoshimura A, Zhdankin V V (2016). Chem Rev.

[32] Quideau S, Pouységu L, Deffieux D (2008). Synlett.

[33] Uyanik M, Ishihara K (2009). Chem Commun.

[34] Liang H, Ciufolini M A (2010). Tetrahedron.

[35] Satam V, Harad A, Rajule R, Pati H (2010). Tetrahedron.

[36] Guérard K C, Sabot C, Beaulieu M-A, Giroux M-A, Canesi S (2010). Tetrahedron.

[37] Singh F V, Wirth T (2011). Org Lett.

[38] Mangaonkar S R, Kole P B, Singh F V (2018). Synlett.

[39] Merritt E A, Olofsson B (2011). Synthesis.

[40] Tinnis F, Stridfeldt E, Lundberg H, Adolfsson H, Olofsson B (2015). Org Lett.

[41] Ghosh R, Stridfeldt E, Olofsson B (2014). Chem – Eur J.

[42] Ghosh R, Lindstedt E, Jalalian N, Olofsson B (2014). ChemistryOpen.

[43] Brown M, Delorme M, Malmedy F, Malmgren J, Olofsson B, Wirth T (2015). Synlett.

[44] Parra A, Reboredo S (2013). Chem – Eur J.

[45] Wang H, Fan R (2010). J Org Chem.

[46] Moriarty R M, Tyagi S, Kinch M (2010). Tetrahedron.

[47] Bose D S, Idrees M (2010). Synthesis.

[48] Pardo L M, Tellitu I, Domínguez E (2010). Synthesis.

[49] Du X, Chen H, Chen Y, Chen J, Liu Y (2011). Synlett.

[50] Wardrop D J, Yermolina M V, Bowen E G (2012). Synthesis.

[51] Singh F V, Wirth T (2012). Synthesis.

[52] Paz N R, Santana A G, Francisco C G, Suárez E, González C C (2012). Org Lett.

[53] Kajiyama D, Saitoh T, Yamaguchi S, Nishiyama S (2012). Synthesis.

[54] Hempel C, Weckenmann N M, Maichle-Moessmer C, Nachtsheim B J (2012). Org Biomol Chem.

[55] Mizar P, Wirth T (2014). Angew Chem.

[56] Qian G, Liu B, Tan Q, Zhang S, Xu B (2014). Eur J Org Chem.

[57] Mizar P, Burrelli A, Günther E, Söftje M, Farooq U, Wirth T (2014). Chem – Eur J.

[58] Yoshimura A, Koski S R, Fuchs J M, Saito A, Nemykin V N, Zhdankin V V (2015). Chem – Eur J.

[59] Mizar P, Niebuhr R, Hutchings M, Farooq U, Wirth T (2016). Chem – Eur J.

[60] Singh F V, Wirth T (2013). Synthesis.

[61] Brown M, Kumar R, Rehbein J, Wirth T (2016). Chem – Eur J.

[62] Malmedy F, Wirth T (2016). Chem – Eur J.

[63] Maertens G, L'Homme C, Canesi S (2015). Front Chem (Lausanne, Switz).

[64] Yeung C S, Dong V M (2011). Chem Rev.

[65] Tamura Y, Yakura T, Haruta J, Kita Y (1987). J Org Chem.

[66] Rama Krishna K V, Sujatha K, Kapil R S (1990). Tetrahedron Lett.

[67] Kita Y, Tohma H, Kikuchi K, Inagaki M, Yakura T (1991). J Org Chem.

[68] Wipf P, Kim Y (1993). J Org Chem.

[69] Ficht S, Mülbaier M, Giannis A (2001). Tetrahedron.

[70] Fujioka H, Komatsu H, Nakamura T, Miyoshi A, Hata K, Ganesh J, Murai K, Kita Y (2010). Chem Commun.

[71] Dohi T, Nakae T, Ishikado Y, Kato D, Kita Y (2011). Org Biomol Chem.

[72] Dohi T, Uchiyama T, Yamashita D, Washimi N, Kita Y (2011). Tetrahedron Lett.

[73] Moschitto M J, Anthony D R, Lewis C A (2015). J Org Chem.

[74] Zhang X, Hou W, Zhang-Negrerie D, Zhao K, Du Y (2015). Org Lett.

[75] Singh F V, Wirth T (2014). Chem – Asian J.

[76] Dohi T, Maruyama A, Yoshimura M, Morimoto K, Tohma H, Kita Y (2005). Angew Chem, Int Ed.

[77] Uyanik M, Yasui T, Ishihara K (2009). Bioorg Med Chem Lett.

[78] Dohi T, Sasa H, Miyazaki K, Fujitake M, Takenaga N, Kita Y (2017). J Org Chem.

[79] Wipf P, Spencer S R (2005). J Am Chem Soc.

[80] Braun N A, Ciufolini M A, Peters K, Peters E-M (1998). Tetrahedron Lett.

[81] Braun N A, Ousmer M, Bray J D, Bouchu D, Peters K, Peters E-M, Ciufolini M A (2000). J Org Chem.

[82] Diaba F, Ricou E, Solé D, Teixidó E, Valls N, Bonjoch J (2007). ARKIVOC.

[83] Wardrop D J, Burge M S, Zhang W, Ortíz J A (2003). Tetrahedron Lett.

[84] Patil A D, Freyer A J, Reichwein R, Carte B, Killmer L B, Faucette L, Johnson R K, Faulkner D J (1997). Tetrahedron Lett.

[85] Honda T (2010). Pure Appl Chem.

[86] Mizutani H, Takayama J, Honda T (2004). Heterocycles.

[87] Wardrop D J, Burge M S (2005). J Org Chem.

[88] Christodoulou M S, Kasiotis K M, Fokialakis N, Tellitu I, Haroutounian S A (2008). Tetrahedron Lett.

[89] Liang J, Chen J, Du F, Zeng X, Li L, Zhang H (2009). Org Lett.

[90] Wang K, Fu X, Liu J, Liang Y, Dong D (2009). Org Lett.

[91] Wang J, Yuan Y, Xiong R, Zhang-Negrerie D, Du Y, Zhao K (2012). Org Lett.

[92] Sreenithya A, Sunoj R B (2014). Org Lett.

[93] Abdellaoui H, Xu J (2014). Tetrahedron.

[94] Southgate R, Branch C, Coulton S, Hunt E, Luckacs G (1993). Recent Progress in the Chemical Synthesis of Antibiotics and Related Microbial Products.

[95] Jin C-Y, Du J-Y, Zeng C, Zhao X-H, Cao Y-X, Zhang X-Z, Lu X-Y, Fan C-A (2014). Adv Synth Catal.

[96] Chen Z-W, Zhu Y-Z, Ou J-W, Wang Y-P, Zheng J-Y (2014). J Org Chem.

[97] Wen J, Wei W, Xue S, Yang D, Lou Y, Gao C, Wang H (2015). J Org Chem.

[98] Dohi T, Maruyama A, Minamitsuji Y, Takenaga N, Kita Y (2007). Chem Commun.

[99] Jaegli S, Dufour J, Wei H-l, Piou T, Duan X-H, Vors J-P, Neuville L, Zhu J (2010). Org Lett.

[100] Dohi T, Takenaga N, Fukushima K-i, Uchiyama T, Kato D, Motoo S, Fujioka H, Kita Y (2010). Chem Commun.

[101] Yu Z, Ju X, Wang J, Yu W (2011). Synthesis.

[102] Zhang D-Y, Xu L, Wu H, Gong L-Z (2015). Chem – Eur J.

[103] Ousmer M, Braun N A, Bavoux C, Perrin M, Ciufolini M A (2001). J Am Chem Soc.

[104] Ousmer M, Braun N A, Ciufolini M A (2001). Org Lett.

[105] Mizutani H, Takayama J, Soeda Y, Honda T (2002). Tetrahedron Lett.

[106] Kita Y, Tohma H, Inagaki M, Hatanaka K, Kikuchi K, Yakura T (1991). Tetrahedron Lett.

[107] Kita Y, Tohma H, Inagaki M, Hatanaka K, Yakura T (1992). J Am Chem Soc.

[108] Kita Y, Takada T, Ibaraki M, Gyoten M, Mihara S, Fujita S, Tohma H (1996). J Org Chem.

[109] Asmanidou A, Papoutsis I, Spyroudis S, Varvoglis A (2000). Molecules.

[110] Zheng C, Wang L, Li J, Wang L, Wang D Z (2013). Org Lett.

[111] Huang J, Wang H, Wu C, Wulff W D (2007). Org Lett.

[112] Phipps R J, Toste F D (2013). J Am Chem Soc.

[113] Tohma H, Harayama Y, Hashizume M, Iwata M, Kiyono Y, Egi M, Kita Y (2003). J Am Chem Soc.

[114] Kita Y, Yakura T, Tohma H, Kikuchi K, Tamura Y (1989). Tetrahedron Lett.

[115] Shigehisa H, Takayama J, Honda T (2006). Tetrahedron Lett.

[116] Honda T, Shigehisa H (2006). Org Lett.

[117] Dohi T, Minamitsuji Y, Maruyama A, Hirose S, Kita Y (2008). Org Lett.

[118] Kita Y, Takada T, Gyoten M, Tohma H, Zenk M H, Eichhorn J (1996). J Org Chem.

[119] Kita Y, Arisawa M, Gyoten M, Nakajika M, Hamada R, Tohma H, Takada T (1998). J Org Chem.

[120] Roe C, Stephenson G R (2008). Org Lett.

[121] Wada Y, Otani K, Endo N, Harayama Y, Kamimura D, Yoshida M, Fujioka H, Kita Y (2009). Org Lett.

[122] Harayama Y, Kita Y (2005). Curr Org Chem.

[123] Canesi S, Belmont P, Bouchu D, Rousset L, Ciufolini M A (2002). Tetrahedron Lett.

[124] Jain N, Ciufolini M A (2015). Synlett.

[125] Shirley H J, Bray C D (2016). Eur J Org Chem.

[126] Uyanik M, Sasakura N, Mizuno M, Ishihara K (2017). ACS Catal.

[127] Ley S V, Schucht O, Thomas A W, Murray P J (1999). J Chem Soc, Perkin Trans 1.

[128] Wipf P, Jung J-K, Rodríguez S, Lazo J S (2001). Tetrahedron.

[129] Wipf P, Lynch S M, Birmingham A, Tamayo G, Jiménez A, Campos N, Powis G (2004). Org Biomol Chem.

[130] Baxendale I R, Ley S V, Nessi M, Piutti C (2002). Tetrahedron Lett.

[131] Quideau S, Lebon M, Lamidey A-M (2002). Org Lett.

[132] El Sayed K A, Bartyzel P, Shen X, Perry T L, Zjawiony J K, Hamman M T (2000). Tetrahedron.

[133] Falomir E, Álvarez-Bercedo P, Carda M, Marco J A (2005). Tetrahedron Lett.

[134] Peuchmaur M, Wong Y-S (2007). J Org Chem.

[135] Wong Y-S (2002). Chem Commun.

[136] Álvarez-Bercedo P, Falomir E, Carda M, Marco J A (2006). Tetrahedron.

[137] Baxendale I R, Deeley J, Griffiths-Jones C M, Ley S V, Saaby S, Tranmer G K (2006). Chem Commun.

[138] Lalic G, Corey E J (2007). Org Lett.

[139] Pouységu L, Sylla T, Garnier T, Rojas L, Charris J, Deffieux D, Quideau S (2010). Tetrahedron.

[140] Koag M, Lee S (2011). Org Lett.

